# An integrative transcriptome study reveals Ddit4/Redd1 as a key regulator of cancer cachexia in rodent models

**DOI:** 10.1038/s41419-021-03932-0

**Published:** 2021-06-26

**Authors:** Mengyuan Niu, Li Li, Zhonglan Su, Lulu Wei, Wenyuan Pu, Chen Zhao, Yibing Ding, Junaid Wazir, Wangsen Cao, Shiyu Song, Qian Gao, Hongwei Wang

**Affiliations:** 1 State Key Laboratory of Analytical Chemistry for Life Science, Medical School of Nanjing University, Nanjing, P. R. China; 2 Center for Translational Medicine and Jiangsu Key Laboratory of Molecular Medicine, Medical School of Nanjing University, Nanjing, P. R. China; 3 Department of Dermatology, the First Affiliated Hospital of Nanjing Medical University, Nanjing, P. R. China

**Keywords:** Cancer metabolism, Transcriptomics

## Abstract

Cancer cachexia is a multifactorial metabolic syndrome that causes up to 20% of cancer-related deaths. Muscle atrophy, the hallmark of cancer cachexia, strongly impairs the quality of life of cancer patients; however, the underlying pathological process is still poorly understood. Investigation of the disease pathogenesis largely relies on cachectic mouse models. In our study, the transcriptome of the cachectic gastrocnemius muscle in the C26 xenograft model was integrated and compared with that of 5 more different datasets. The bioinformatic analysis revealed pivotal gene ontology (GO) terms and Kyoto Encyclopedia of Genes and Genomes (KEGG) pathways of the disease, and the key genes were validated. Construction of the protein-protein interaction network and the comparison of pathways enriched in cancer cachexia with 5 other muscle atrophy models revealed Ddit4 (DNA damage-inducible transcript 4), as a key protein in cancer cachexia. The higher expression of Ddit4 in cachectic muscle was further validated in animal models and cachectic cancer patients. Further study revealed that p38 induced the expression of Ddit4, which in turn inhibited the mTOR pathway in atrophic cells.

## Introduction

Cancer cachexia is a multifactorial metabolic syndrome that affects approximately 50–80% of cancer patients, especially in the advanced disease stages [1]. It is characterized by a negative energy balance of reduced food intake and exceeded metabolism and causes up to 20% of cancer deaths [2]. Clinically, cancer cachexia is defined as weight loss of greater than 5% or weight loss greater than 2% in individuals already showing depletion according to current body weight and height (body-mass index [BMI] < 20 kg/m²) or skeletal muscle mass (sarcopenia) within 12 months [3].

Muscle atrophy is the hallmark of cancer cachexia. It causes physical malfunction, compromises the immune system, leads to insulin resistance and fatigue, and strongly impairs patients’ quality of life [4, 5]. Thus, protection from muscle loss is vital in the treatment of cancer cachexia. However, there are no approved, effective treatments for muscle wasting in cancer, and nutrition support alone cannot reverse these symptoms [1]. Therefore, a profound understanding of the alteration in muscle atrophy at the molecular level is urgently needed.

Various rodent models, including cancer cell xenograft models [6, 7], patient-tumor xenograft (PDX) models [8], and transgenic mouse models [9] have been established to mimic the muscle wasting phenotype of cancer cachexia. Although studies of these models have revealed numerous mechanisms of cancer cachexia, an integrative study is still needed, to uncover the possible general molecular pathways and genes in the key organ, muscle, regardless of the model adopted. Furthermore, a comparison of the overall transcription profile between cancer or noncancer-induced muscle atrophy will be greatly helpful in revealing the uniqueness of the disease.

Here, the transcriptome of the cachectic gastrocnemius muscle was compared with that of normal muscle in a C26 xenograft-induced cancer cachectic model. Gene ontology (GO) terms and Kyoto Encyclopedia of Genes and Genomes (KEGG) pathways of differentially expressed genes (DEGs) were enriched. The data were then integrated with 5 published cachectic mouse muscle transcriptome datasets for further investigation. The batch effect of the merged data was removed, and GO term and KEGG pathway enrichment analysis was performed according to the DEGs discovered between cachectic and normal muscle. The merged data differences were compared with our results at the gene and pathway levels, and key genes were validated. In addition, a protein–protein interaction (PPI) network based on the enriched genes was constructed to allocate key gene nodes in cancer cachexia and compared with other muscle atrophy models and the biological function was further confirmed at the cell level, in the animal models, and in cancer cachexia patients.

## Materials and methods

### Cell culture

The colon-26 (C26) murine adenocarcinoma cell line was cultured as a monolayer with RPMI-1640 medium (Life Technology, New York, NY, USA) supplemented with 10% fetal bovine serum (HyClone, Logan, UT, USA), L-glutamine, 100 units/ml penicillin and 100 mg/ml streptomycin (HyClone) in a humidified atmosphere containing 5% CO_2_ at 37 °C. Murine C2C12 myoblasts obtained from the American Type Culture Collection (ATCC) were maintained in Dulbecco’s modified Eagle medium (DMEM) supplemented with 10% fetal bovine serum and antibiotics (100 U/ml penicillin and streptomycin, referred to as growth medium, GM). To induce myotube differentiation, the cells were grown to 100% confluence and exposed to DMEM containing 2% horse serum (Life Technology) (referred to as differentiation medium, DM) for up to 4 days. No mycoplasma contamination was validated by PCR in these cell lines before experiments.

### Experimental mice

All mouse experiments were performed with the approval of the Institutional Animal Care and Use Committee of Nanjing University (IACUC-2003124). Six-week-old male BALB/C mice were purchased from the Model Animal Research Center of Nanjing University, maintained in specific-pathogen-free (SPF) conditions, and acclimated for 1 week. Mice were housed in IVC cages (five per cage) with free access to drinking water and a basal diet under controlled conditions of humidity, light (12 h light/12 h dark cycle), and temperature. The mice were randomly assigned to cachexia or control group (*N* = 10). For the cachexia model generated by tumor cell xenograft, 100 μl C-26 murine adenocarcinoma cells were subcutaneously inoculated into the right flanks of the mice (5 × 10^5^ cells/mouse). Mice were weighed daily and then euthanized under isoflurane anesthesia. Tissues were collected, weighed, snap-frozen in liquid nitrogen for RNA extraction, or fixed in 4% paraformaldehyde for histochemistry analysis.

### Patient samples

The study was approved by the Ethics Committee of Affiliated Drum Tower Hospital, Medical School of Nanjing University (IRB number: 20200115003). All patients provided written informed consent. Cachexia was defined clinically as documented non-intentional dry weight loss of >5 kg (all > 10% of their previous normal weight) in ≤ 3 months [10].

All noncachectic control patients don’t have obvious involuntary weight loss not explained by other diseases or recent surgery. Cachectic patient biopsy specimens of skeletal muscle were collected after hepatobiliary surgery. Noncachectic control samples were acquired from patients undergoing elective orthopedic surgery. Five cachectic and four noncachectic control patients were recruited. All eligible patients gave written informed consent. The demographic information of the patients was listed in Table S1.

### RNA isolation and sequencing

Total RNA was isolated from muscle biopsies using TRIzol (Invitrtogin) according to established methods [11]. The mRNAs were enriched by removing rRNA with RNaseH. Target RNAs were fragmented into short fragments in the fragmentation buffer, and cDNAs were synthesized using the RNA fragments as templates for N6 random primers, followed by end reparation and ligation to adaptors. The quantity and quality of the cDNA libraries were assessed using an Agilent 2100 BioAnalyzer. Finally, the libraries were sequenced on the BGISEQ-500 with 50 single-end reads. Sequencing reads that contained adaptors were of low quality, or that aligned to rRNA were filtered off before mapping. The clean sequence reads were then aligned with the reference genome of Mus musculus (GRCm38.p5) downloaded from NCBI using bowtie2. Fragments per kilobase of transcript per million mapped reads values were obtained by transforming mapped transcript reads using RSEM. The data were submitted to GEO (GSE175570).

### Data processing

The transcription data of the muscles of cachectic mice and other muscle atrophy models were downloaded from the GEO database (GSE numbers: GSE133524 as test1; GSE138464 as test2; GSE107470 as test3, GSE142455 as test4; GSE137985 as test5, the information of the model adopted, the sample numbers, the gene background of mice, tissue collected, et al was listed in Table S4 with detail.) with the “GEOquery” R package [12]. Only samples without any additional treatment were included. Our RNA sequencing result was assigned as test6. Other muscle atrophy models (GSE62897, Limb immobilization; GSE81367, Huntingdon disease; GSE124394, fasting; GSE39195, Denervation; and GSE139213, Rapamycin) were also quired as above. The data in fragments per kilobase of transcript per million mapped reads (FPKM) or count form were transformed to transcripts per million (TPM). The numbers of transcripts validated in each dataset varied from 11,188 to 26,061. To compare the variation between cachectic conditions in all datasets, a total of 9964 transcripts shared in all datasets were addressed. The expression values not in log form were log2‐transformed before cross‐platform normalization. The bath effect of the datasets was removed by the “sva” package [13]. Based on the systematic evaluation, the ComBat function was applied to the data to remove the batch effect between the platforms [14]. Principal component and clustering analysis were used to evaluate whether the batch effect was removed. The data were then normalized by TMM method [15], and the DEGs were identified between cachectic mice and control ones with the “limma” R package [16].

### Functional enrichment analysis

The KEGG pathway and GO biological process enrichment were analyzed, and visualized by the “clusterProfiler” R package [17] or GSEA software(Version 4.1.0 [18]). As the GSEA analysis was based on human genes, the gene symbols of mouse were transformed to human homologs by biomaRt R package [19] before enrichment.

### Identification and validation of hub genes

For biological interactions, the genes were submitted to the Search Tool for the Retrieval of Interacting Genes (STRING, http://www.string-db.org/ [20]), and the PPI network was retrieved, analyzed, and visualized by Cytoscape v3.7.2 software [21]. The color of the node was mapped to the degree of the gene, and the circle and font size of the node was mapped to the number of direct edges.

### Quantitative PCR

Total RNA from snap-frozen muscle was isolated following the RNeasy Micro Kit protocol (Qiagen, Hilden, Germany). The mRNA was reverse-transcribed into cDNA with PrimeScript RT Master Mix (TaKaRa, Otsu, Japan) and subjected to quantitative real-time PCR with SYBR Green using PCR Master Mix (Life Technology). Primer sequences of all tested genes are provided in Table S2.

### Transfection of small interfering RNAs (siRNAs) and overexpression plasmids

siRNA transfection was performed using Lipofectamine RNAiMAX transfection reagent (Life Technology) by following the manufacturer’s guidelines. Briefly, C2C12 cells were seeded into a six-well plate. After four days of differentiation, 5 pmol of siRNA was diluted with Opti-MEM and mixed with the transfection reagent, and the mixture was added to each well. After transfection for 24 h, the cells were challenged with different treatments; some were co-cultured with C26 cell-conditioned media (C26 CM), and some were cultured in DMEM. The siRNA sequences specific for Ddit4 are listed in Table S2.

For Ddit4 overexpression, the ORF of Ddit4 was synthesized and cloned to pcDNA3.1 vector by Genescript (Nanjing, China).

C2C12 cells were transiently transfected with Ddit4 plasmids using the Lipofectamine 2000 Transfection Reagent according to the manufacturer’s instructions (Life Technology).

### Histology and immunofluorescence

Procedures for determining the fiber cross-sectional area (CSA) were conducted as previously described [22]. Briefly, fixed transverse sections (7 μm) were cut from the mid-belly of the gastrocnemius with a cryostat at −20 °C and then stained with hematoxylin and eosin (H&E). The CSA and number of myofibers with central nuclei among the individual myofibers and the diameter of the myotubes were determined by using Fiji software [23].

For immunofluorescence analysis, cells were seeded onto sterile preprocessed glass coverslips that were precoated with 1% gelatin. After C2C12 myoblasts had differentiated into myotubes, the cells were washed twice with PBS, followed by fixation in 4% paraformaldehyde for 15 min. After being rehydrated in PBS, the cells were blocked for 30 min in 1% bovine serum albumin (BSA) in PBS containing 0.2% Triton-X (PBST). Then, the cells were incubated with an anti-myosin heavy chain (MHC) (R&D Systems, Minneapolis, MN, USA) (1:100) in 1% BSA/PBST overnight at 4 °C. Cells were then incubated with a fluorescence-labeled secondary anti-mouse antibody (1:1000) and DAPI (1:1000) at room temperature for 1 h. The specimens were examined under an FV10i laser scanning confocal microscope (Olympus, Tokyo, Japan). The fluorescence score was calculated by multiplying the average light intensity and area of fluorescence staining with Fiji software.

### Western blotting

Total protein extract was obtained by homogenizing either skeletal muscle or C2C12 myotube samples in RIPA buffer (150 mM NaCl, 1.0% NP-40, 0.5% sodium deoxycholate, 0.1% SDS, and 50 mM Tris, pH 8.0) supplemented with a protease inhibitor cocktail (Roche, Indianapolis, IN, USA). The protein concentration in the supernatants was determined with a BCA kit (Pierce, Rockford, IL, USA). Then, 20 µg of total protein was separated by electrophoresis in a 10% SDS-polyacrylamide gel and transferred to a PVDF membrane (Millipore, Billerica, MA, USA) for western blot analysis. Antibodies against p70S6K1 (Thr389), 4E-BP1 (Ser65), mTOR, mTOR (Ser2448), p-P38, P38, and p62 were obtained from Cell Signaling Technology (Danvers, MA, USA). Antibodies against total p70S6K1 and 4E-BP1 were obtained from Beyotime (Nanjing, China). Antibodies against Ddit4, LC3, and GAPDH were obtained from ProteinTech. P38 inhibitor-SB203580 was purchased from Beyotime (Nanjing, China).

### Statistical analysis

Data were presented as the mean ± SD. For comparisons of two groups that were normally distributed, the student’s *t*-test was adopted. The Mann-Whitney’s rank-sum test was used for the values that were not normally distributed (as determined by the Kolmogorov–Smirnov test). For comparisons of more than two groups, analysis of variances was adopted (ANOVA). A *P* value less than 0.05 was deemed statistically significant. All statistical tests were two-sided and were performed using GraphPad Prism 8 (GraphPad Software, La Jolla, CA, USA).

## Results

### Characterization of the colon-26 cancer cachexia model

**Fig. 1:**
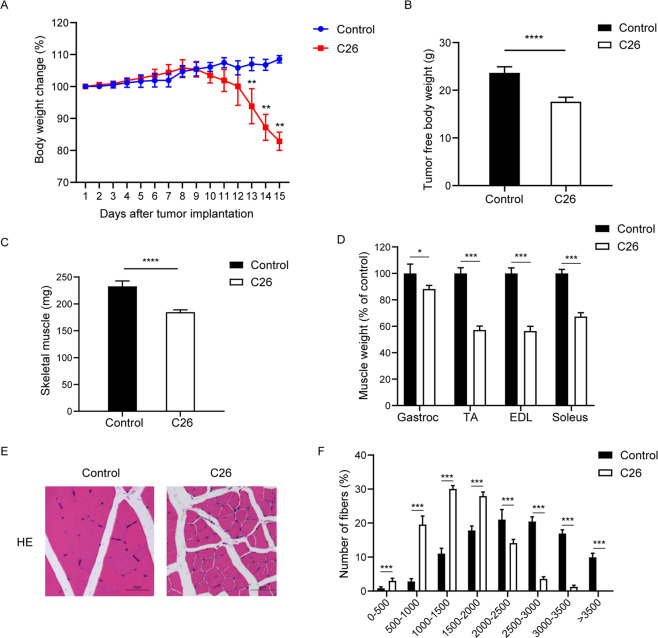
Characterization of the colon-26 cancer cachexia model. **A** Bodyweight changes in normal mice and C26 tumor-bearing mice were monitored every day and traced for 15 days after tumor implantation. **B** Tumor-free body weights were measured after removal of the tumor at the end of the experiment. **C** The weight of total skeletal muscle of C26-xenografted mice was compared with that of normal control. **D** The weights of gastrocnemius (gastroc), tibialis anterior (TA), extensor digitorum longus (EDL), and soleus of C26-xenografted mice vs. nontumor-bearing control mice. **E** Representative haematoxylin and eosin (H&E) staining showing the morphological changes in the gastrocnemius of C26-xenografted and control mice. Scale bar: 50 μm. **F** Fiber area frequency distribution of all fibers from gastrocnemius muscles of control and C26-xenografted mice. Data are expressed as the mean ± SD, *P < 0.05, **P < 0.01, ***P < 0.001, ****P < 0.0001, *N* = 10.

As previously described, inoculation of C26 adenocarcinoma cells into male Balb/C mice is a classic model of cancer cachexia [6, 24]. In our experiment, C26-xenografted mice manifested cachexia wasting syndrome characterized by progressive body weight loss (~20% at 15 days post C26 cell inoculation) and a reduction of skeletal muscle (20.7% vs. control) (Fig. 1A–C). The weight of the gastrocnemius (gastroc) decreased by 11.8%; that of the tibialis anterior (TA) decreased by 42.8%; that of the extensor digitorum longus (EDL) decreased by 43.6%, and that of the soleus decreased by 32.7% compared with the nontumor-bearing control mice (Fig. 1D). Morphometric analysis of muscle CSA revealed that the fiber diameters of C26 mice were markedly reduced compared with those of noninoculated control mice (Fig. 1E and F).

### Transcriptome analysis of skeletal muscle discriminates cachectic vs. noncachectic mice

**Fig. 2:**
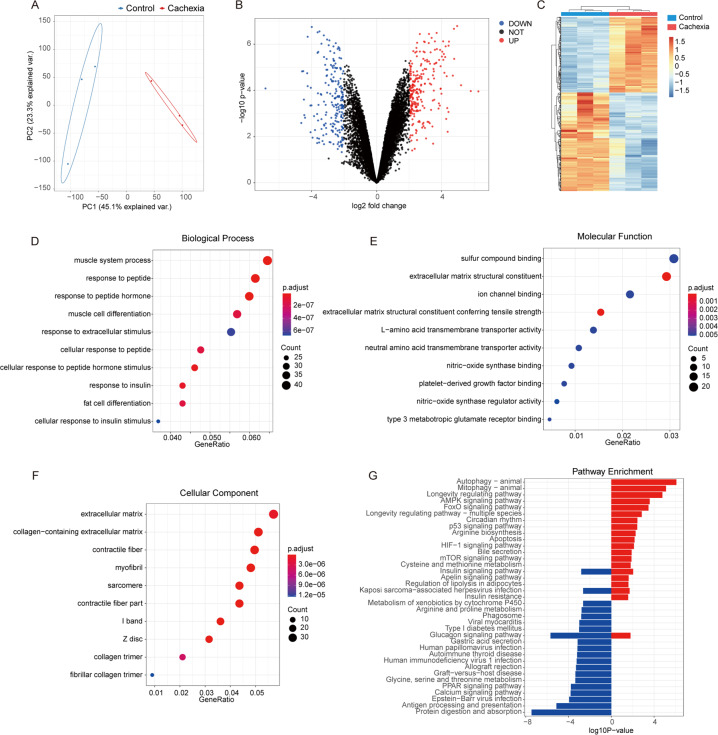
RNA sequencing effectively discriminates between cachectic and noncachectic mice. **A** Principal component analysis (PCA) of the transcriptome of the muscles of C26-xenografted and control mice. **B** Volcano plots of differentially expressed genes (DEGs) of atrophied muscles with normal ones. Red dots represent upregulated DEGs (192), and blue dots represent downregulated DEGs (254). **C** Hierarchical clustering heatmap of the profile of total DEGs between atrophied muscles and normal muscles. **D–F**. Gene ontology (GO) enrichment analysis of DEGs in biological process (**D**) molecular function (**E**) and cellular component (**F**) categories. **G** KEGG pathway mapping of DEGs. Red indicates upregulated pathways, blue indicates downregulated pathways.

We analyzed the transcriptome of the atrophied muscles using RNA sequencing to identify the differences in expressed genes associated with the cancer-induced wasting phenotype. A total of 18,464 transcripts were identified after pre-cleaning the raw sequencing data. Unsupervised principal component analysis (PCA) revealed that the gene expression profiles of atrophied muscles were significantly separated from normal controls (Fig. 2A). The volcano plot showed the distribution of the DEGs according to the criteria of adjusted *P* value < 0.05 and an absolute value of fold change > 4; the red dots represented upregulated DEGs (192), and the blue dots represented downregulated DEGs (254) (Fig. 2B). A hierarchical clustering heatmap showed that the profile of the relative expression of all DEGs in cachectic samples was distinct from that of the normal samples (Fig. 2C). To explore the biological functions of the DEGs, GO enrichment analysis was performed. There was significant enrichment of GO terms including the biological processes of the fatty acid metabolic process (GO: 0006631), fat cell differentiation (GO: 0045444), muscle system process (GO: 0003012). (Fig. 2D); enrichment in molecular function terms of extracellular matrix structural constituent (GO: 0005201), platelet-derived growth factor binding (GO: 0048407), ion channel binding (GO: 0044325), etc. (Fig. 2E); and enrichment in cellular component terms of extracellular matrix (GO: GO: 0031012), collagen-containing extracellular matrix (GO: 0062023), and contractile fiber (GO: 0043292) (Fig. 2F). Mapping of the DEGs to KEGG pathways showed that the pathways of mitophagy-animal (mmu04137), autophagy-animal (mmu04140), and FoxO signaling (mmu04068), etc. were significantly enriched in upregulated genes, while protein digestion and absorption (mmu03320), PPAR signaling (mmu03320), and autoimmune thyroid disease (mmu05320), etc. were significantly enriched in the downregulated genes. (Fig. 2G). The enrichment results indicated an alteration of metabolism, enhanced autophagy, and signal transduction of FoxO signaling in the atrophied muscles. The full enrichment results of GO terms and KEGG pathways are listed in Table S3.

### Removal of the batch effect by cross-platform normalization for integrative investigation

To further explore the significance of the C26 model and the possible general underlying mechanisms of cachexia, 5 more RNA sequencing datasets of muscles of different cachectic mouse models, including allogeneic transplantation of mouse tumor, patient-derived tumor xenograft, and transgenic cancer models, were downloaded from the GEO database. The datasets were assigned as test1 to test5 and initially merged with our results (test6) according to a total of 9964 transcripts shared in all datasets. Only samples from limb muscle without additional treatment were included. A detailed description of all datasets is listed in Table S4.

**Fig. 3:**
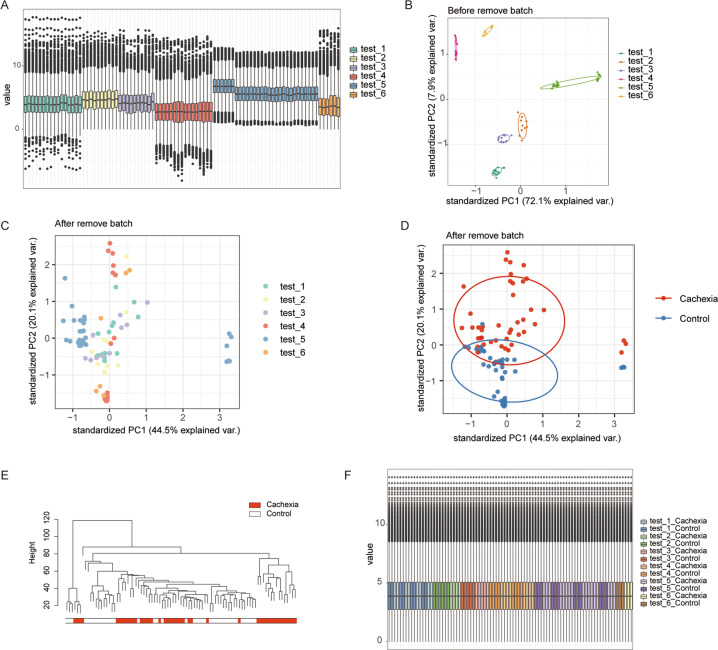
Removal of batch effect between different cachexia mouse models. **A** The boxplot shows the profile of the distribution of transcript levels in each dataset. **B** PCA of 6 datasets before the removal of the batch effect. **C**, **D** PCA of the merged dataset after the removal of the batch effect. (**C**) the color of the samples was mapped to the data source, (**D**) the color of the samples was mapped to the cachectic condition. **E** Hierarchical clustering assay of the transcriptome of the merged data; the samples were grouped according to the cachectic condition. **F** The boxplot shows the overall transcript expression profile of each dataset.

As shown in Fig. 3A, the profile of the transcripts varied significantly between each test, and the samples were clustered corresponding to the source of tests both in PCA and in hierarchical clustering assays (Fig. 3B, Fig. S1A), suggesting a strong batch effect of the data. Thus, we evaluated and removed the batch effect of the data by surrogate variable analysis with the ‘sva’ R package. As shown in Fig. 3C and D, after removing the batch effect, the samples showed a significant clustering effect according to the cachectic condition but not the tests. Hierarchical clustering assays also confirmed that the samples showed significant differences between cachectic tissue and normal tissues at the transcriptional profile level (Fig. 3E). After further normalization between each sample, the data showed good expression consistency between samples, indicating that they were suitable for further DEG identification (Fig. 3F).

### Identification and biological function enrichment of DEGs of the muscle between all cachectic and normal mice

**Fig. 4:**
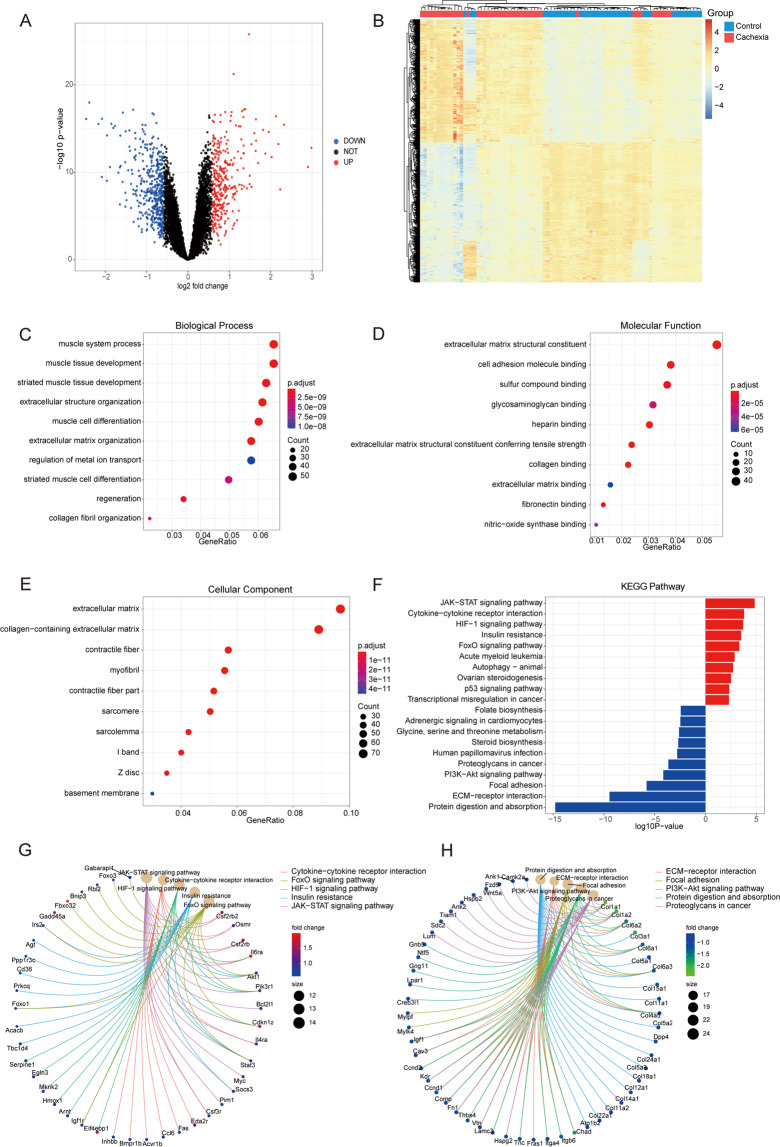
Identification of DEGs and biological function enrichment analysis in the merged dataset. **A** Volcano plots of DEGs in the merged dataset. Red dots represent upregulated DEGs (371), and blue dots represent downregulated DEGs (422). **B** Heatmap of the DEGs showing a significant distinction between cachectic mice and control mice. **C–E**. GO enrichment analysis of DEGs in terms of biological process (**C**), molecular function (**D**), and cellular component (**E**)**. F** KEGG pathway mapping of DEGs. Red indicates upregulated pathways, blue indicates downregulated pathways. **G**, **H**. The circle map depicts the linkages of genes and KEGG pathways of upregulated (**G**) and downregulated (**H**) genes in cachectic muscle compared with control muscle.

Next, the processed dataset was analyzed to identify the DEGs across all the datasets. Transcripts that had an adjusted *P* value < 0.05 and an absolute value of fold change > 1.5 were considered as DEGs. The volcano plot showed the distribution of the DEGs according to the criteria; red dots represented upregulated DEGs (371), and blue dots represented downregulated DEGs (422) (Fig. 4A). The heatmap of the DEGs shows that the transcriptional profile of cachectic mice is distinct from that of control mice (Fig. 4B).

The GO term enrichment analysis indicated the enrichment of extracellular matrix organization (GO: 0030198), extracellular structure organization (GO: 0043062), muscle system process (GO: 0003012), muscle cell differentiation (GO: 0042692), and muscle tissue development (GO: 0060537) in the category of biological process (Fig. 4C); extracellular matrix structural constituent (GO: 0005201), extracellular matrix structural constituent conferring tensile strength (GO: 0030020), collagen binding (GO: 0005518), heparin-binding (GO: 0008201), and cell adhesion molecule binding (GO: 0050839) in the category of molecular function (Fig. 4D); and collagen-containing extracellular matrix (GO: 0062023), extracellular matrix (GO: 0031012) and myofibril (GO: 0030016) in the cellular component category (Fig. 4E).

The search and mapping of the genes to the KEGG pathways showed that JAK-STAT signaling (mmu04630), cytokine-cytokine receptor interaction (mmu04060), HIF-1 signaling (mmu04066), insulin resistance (mmu04931), and FoxO signaling (mmu04068) were significantly enriched among the upregulated genes. Protein digestion and absorption (mmu04974), ECM-receptor interaction (mmu04512), focal adhesion (mmu04510), and PI3K-AKT signaling (mmu04151) were significantly enriched among the downregulated genes (Fig. 4F). Similar results were acquired from GSEA analysis of the human homolog of the genes (Fig. S1B). And, the genes enriched in the corresponding pathways were highlighted by red (upregulated) or green (downregulated) (Fig. S2).

To further address the potential biological complexities of the crosstalk of key genes in the pathways, we depicted the linkages of genes and KEGG pathways as circle networks (Fig. 4G–H). Among the upregulated genes, Il6ra, Cdkn1a, Csf2rb2, and Akt1, and among the downregulated genes, Col1a1 and other collagen-related genes were involved in multiple pathways, suggesting their key function in cachexia.

### Comparison of the C26 model with integrative data and validation of key genes

**Fig. 5:**
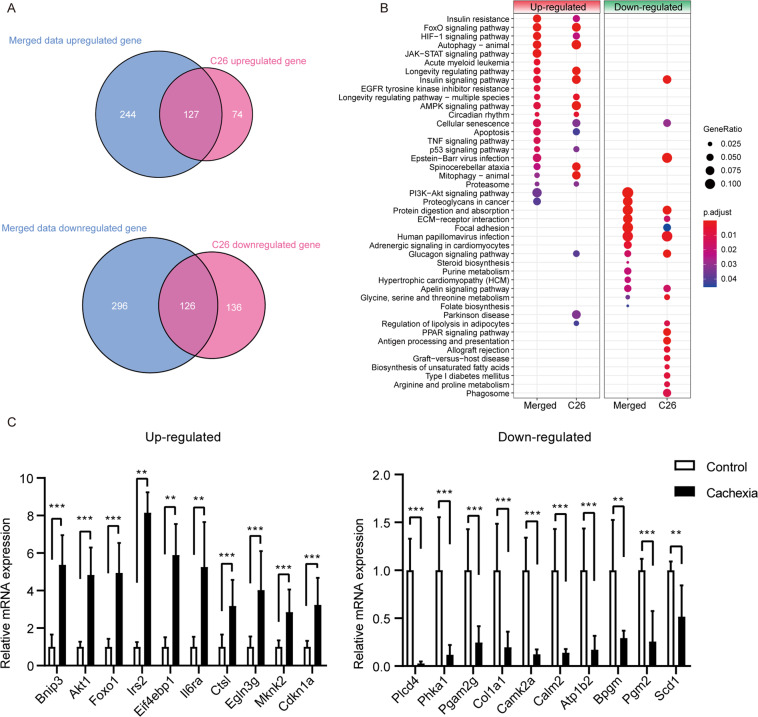
Comparison of the C26 model with integrative data and gene validation. **A** The Venn plot shows the overlap of 127 upregulated DEGs and 126 downregulated ‘DEGs in the C26 model with merged data. **B** The top 25 predominately enriched pathways of upregulated and downregulated genes in the merged dataset and C26 dataset were compared. **C** Validation of the upregulated and downregulated DEGs in the enriched KEGG pathways in the merged dataset was performed by qPCR. Data are expressed as the mean ± SD, *P < 0.05, **P < 0.01, ***P < 0.001, ****P < 0.0001, *N* = 10.

Next, we compared the DEGs and pathways that were significantly enriched in the C26 model with those in the merged data. As shown in Fig. 5A, the top half of the DEGs in the C26 model overlapped with the merged data, suggesting that the C26 model may partially represent the transcript profile of the muscle that underwent cachectic challenge. Interestingly, at the pathway level, there were significant overlapping pathways that were predominately enriched in each dataset, such as the upregulation of mitophagy, autophagy, and FoxO signaling pathways and downregulation of protein digestion and absorption, glycine, serine and threonine metabolism, glucagon signaling and ECM-receptor interaction. Moreover, the JAK-STAT signaling, PI3K-AKT signaling, proteoglycans in cancer, and focal adhesion pathways were enriched in the merged data but not in the C26 model. In contrast, the PPAR signaling and antigen processing and presentation pathways were enriched only in the C26 model (Fig. 5B).

Thus, we tried to validate some shared key genes at the transcriptional level. Genes that were upregulated and represented in the overlap at both the gene and pathway levels of the C26 and merged data, such as Bnip3, Akt1, Foxo1, Irs2, Eif4ebp1, Il6ra, Ctsl, Egln3g, Mknk2, and Cdkn1a, were validated by RT-qPCR. The same test was also performed with downregulated genes such as Plcd4, Pka1, Pgam2, Col1a1, Camk2a, Atp1b2, Bpgm, Pgm2, and Scd1 (Fig. 5C). The validation showed good consistency of the qPCR results and RNA sequencing results.

### Construction of the protein–protein interaction network and comparison of cancer cachexia with other muscle atrophy models revealed Ddit4 as a key protein in cachexia

**Fig. 6:**
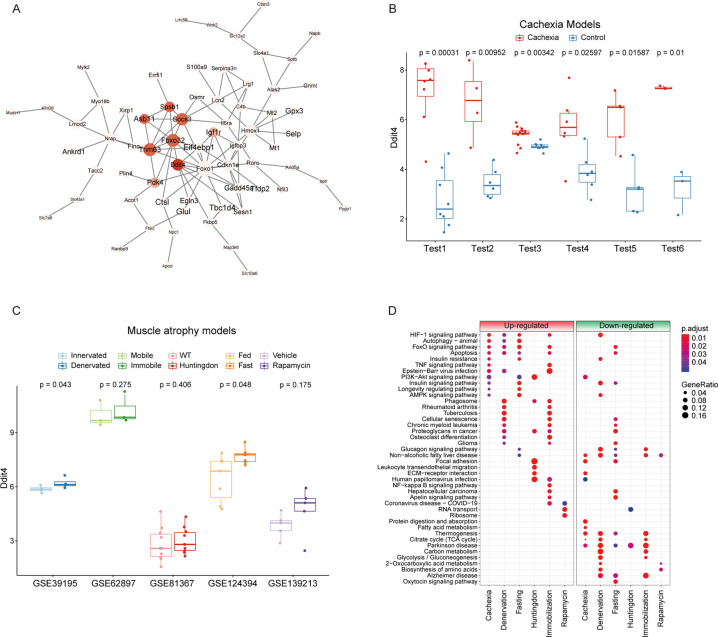
The protein interaction network revealed Ddit4 as a key protein in cancer cachexia. **A** The protein-protein interaction (PPI) network of upregulated genes in the enriched KEGG pathways was constructed according to their biological association. The color of the node indicates the degree of the gene, and the circle and font size of the node indicates the number of direct edges. **B** The expression of Ddit4 in each cachexia model. **C** The expression of Ddit4 in other muscle atrophy models such as denervated, fasting, immobilization, Huntingdon disease, and rapamycin-treated mice. **D** All enriched pathways of upregulated and downregulated genes in cachexia and degenerative conditions such as fasting and denervation were compared.

To further construct the biological gene network and allocate node genes in the cachectic muscle, the genes significantly enriched in KEGG pathways were submitted to the STRING database. The upregulated genes presented a complicated network that was composed of multiple key factors that were reported to be involved in the process of the cachectic phenotype in the muscle, such as Fbxo32, FoxO1, and members of the Stat3 and/or Akt1 pathway (Fig. 6A). The network of downregulated genes is presented in Fig. S3. Notably, the gene of Ddit4 was located at the center and had a large degree of connection with neighbor nodes, indicating its high position in the network (Fig. 6A). Furthermore, it also had a high fold change (> 4.50) and strong significance (*p* < 4.8 × 10^11^) between the cachexia group and the control group. The expression of Ddit4 was significantly different across each cachexia model (Fig. 6B), and genes biologically related to Ddit4 were also significantly different between the muscles of normal and cachectic mice (Fig. S4).

Thus, we were wondering whether Ddit4 is acting as a general player in other muscle atrophy models. Not as expected, unlike in cancer cachexia models, Ddit4 showed a mild upregulation in the muscles in denervated and fasting models but not significantly altered in other muscle atrophy models such as immobilization, Huntingdon disease, and rapamycin-treated mice (Fig. 6C). What’s interesting is that on the one hand, there were no genes that were shared across all these models (Fig. S5); on the other hand, at the pathway level, the sharing pathways as HIF-1 signaling, Autophagy, FoxO signaling between cachexia and degenerative conditions such as fasting and denervation, suggesting different molecules were adopted in different diseases to achieve the atrophy consequence upon these disorders (Fig. 6D). Thus, it seems that Ddit4 played a unique and pivotal role in cancer cachexia.

### p38 induced Ddit4 upregulation inhibited mTOR activation in myotube atrophy

Since Ddit4 is known to function as an inhibitor of mammalian target of rapamycin (mTOR) protein kinase in complex 1 (mTORC1) in response to diverse stress conditions [25, 26]. And the genes involved in the mTOR pathway were significantly altered in cancer cachectic muscles (Fig. S4). Thus, the expression of Ddit4 and mTOR signaling pathway-related proteins were then validated in the C26 model.

**Fig. 7:**
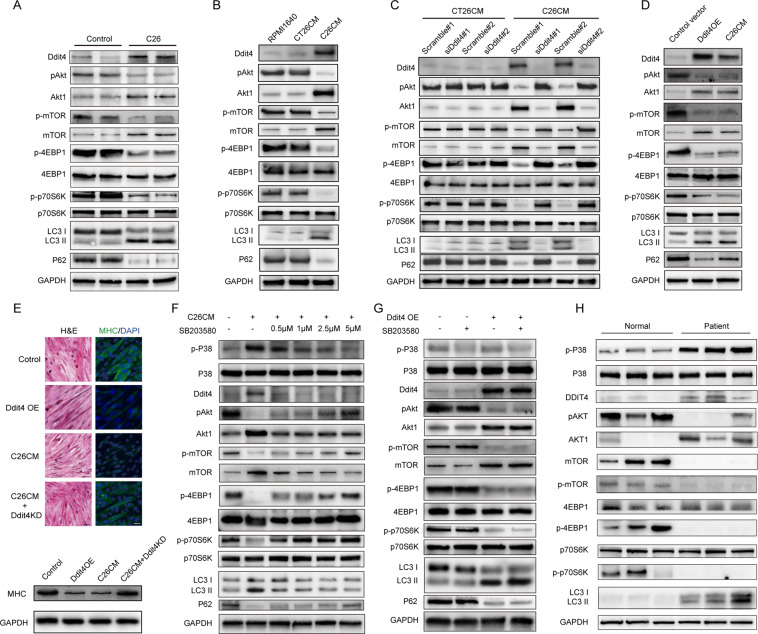
Knocking down of Ddit4 in C2C12 reversed abnormal mTOR signaling and attenuated autophagy. **A** Western blot analysis of the expression of the indicated muscle-related genes between normal and cachectic mice. **B** C2C12 myotubes were treated with RPMI1640, CT26 CM, and C26 CM. Western blot analysis of the indicated genes were detected. **C** C2C12 myotubes were treated with CT26 CM, CT26 CM plus Ddit4 siRNA (two different siRNA of Ddit4 named #1 and #2) or scramble siRNA, C26 CM, C26 CM plus Ddit4 siRNA (two different siRNA of Ddit4 named #1 and #2) or scramble siRNA for 24 h. Western blot analysis of the indicated genes in C2C12 cells with Ddit4 knockdown and/or C26 CM challenge. **D** C2C12 myotubes were treated with control vector, Ddit4 overexpression plasmid, or C26 CM for 24 h. **E** Upper panel: the morphological changes in C2C12 myotubes were addressed by H&E staining (left panel). C2C12 cells were immunostained with the MHC (MF20) antibody. MHC, green, DAPI, blue (right panel). Scale bar: 50 μm. Lower panel: Western blot analysis of MHC in C2C12 myotubes. **F** C2C12 cells were pretreated with (+) or without (−) SB203580(0.5, 1, 2.5, and 5 µM) prior to C26 CM for 24 h. Cell lysates were subjected to Western blot analysis; **G** C2C12 cells were pre-treated with (+) or without (−) SB203580 (5 µM) and then transfected with Ddit4-overexpressing plasmid. Cell lysates were subjected to Western blot analysis. **H** Western blot detection of the indicated genes in the muscle of healthy people and the muscle of liver cancer patients with cachexia.

In C26 model mouse muscles, the expression of Ddit4 was upregulated, while phosphorylated forms of Akt1, mTOR, phospho-4E-BP1, and phospho-p70S6K were downregulated, suggesting an insulin resistance-like phenotype (Fig. 7A). However, inconsistent with the R-seq data, the total protein levels of Akt1 and mTOR were upregulated, which might be a compensation for the blockage of this pathway (Fig. 7A). We then adopted the in vitro cachectic cell model by culturing the C2C12 myotubes with a C26 conditional medium (C26 CM). As shown in Fig. 7B, C26 CM upregulated Ddit4, Akt1, and mTOR expression and downregulated phospho-4E-BP1, and phospho-p70S6K in C2C12 compared with CM of CT26, another colon cancer cell line that could not lead to cancer cachexia. CT26 CM could serve as a negative control. And overexpressing Ddit4 in C2C12 cells suppressed Akt1 phosphorylation and downstream genes and increased cell autophagy as verified by the level of LC3 and p62, mimicking the effect of C26 CM (Fig. 7D). In contrast, knocking down Ddit4 in C2C12 cells restored the levels of phospho-Akt1 and phospho-mTOR, attenuated autophagy, and reversed the C2C12 myotube shrinking and MHC (myosin heavy chain) loss induced by C26-conditioned medium (CM) validated by immunofluorescence, western blot, and qPCR (Fig. 7C and E, Fig. S6A and B).

As the p38 mitogen-activated protein kinase (MAPK) activation is the most well-known mechanism in inflammatory conditions and oxidative stress, including cancer cachexia [27–29], the pretreatment of SB203580 (an inhibitor of p38 MAPK) significantly inhibited p38 phosphorylation, downregulated Ddit4 expression and reversed autophagy in a dose-dependent manner in C2C12 cell challenged with C26 CM (Fig. 7F). Furthermore, qPCR reveals Ddit4 was also downregulated by p38 inhibition at the transcription level (Fig. S6C). And the effect of SB203580 was abolished by the overexpression of Ddit4 (Fig. 7G), suggesting that Ddit4 was downstream of p38 regulation.

To expand the clinical significance of the finding, the muscles of liver cancer cachexia patients and normal people were analyzed. As shown in Fig. 7H, the level of Ddit4 and autophagy-related genes was significantly higher in the muscles of cachectic patients. At the same time, the phosphorylation of Akt1 and mTOR was lower, which was consistent with our in vitro results. Thus, we might conclude that the p38-Ddit4-mTOR axis is crucial in the context of muscle atrophy in cancer cachexia.

## Discussion

Cancer cachexia is a severe condition that occurs in 50–80% of cancer patients, especially in the late stage [2]. There is still no efficient treatment for it. One of the major obstacles to investigating cancer cachexia in clinical practice is the difficulty of collecting tissues, especially muscle, from cachectic cancer patients [30]. Thus, until now, the greatest advancements in understanding this disease and developing therapy were based on animal models. A large number of valuable mechanisms were obtained from rodent cachectic model studies [6, 8, 31]. Overexpression of the E3 ubiquitin-protein ligase of Trim63 (also known as Murf1) and F-box only protein 32 (Fbxo32, also known as Atrogin1) [32, 33] enhances the ubiquitin-dependent protein degradation of muscle component proteins in atrophic muscles. The expression of these elements is under the control of the transcription factors forkhead box protein O1 (Foxo1) and Foxo3, which act as regulatory nodes between anabolic and catabolic processes [34, 35]. Moreover, AKT activity is critical for protein synthesis and cell survival in muscle cells, which is often suppressed in cancer cachexia. Furthermore, the inhibition of AKT activates cell autophagy by increasing the expression of Beclin 1, autophagy protein 5, and microtubule-associated protein 1A/1B light chain 3B (MAP1LC3B) [36].

The differences between the models adopted, e.g., the source of the tumor, the type of tumor cell line, the site of tumor plantation, the immune condition, and the genetic background of the mouse strain, all might influence the mechanism underlying the cachectic condition. On the other hand, all the muscles were considered a cachectic phenomenon by physical tests and the pathological assessment of the muscle section. Thus, we hypothesized that a general mechanism underlying cancer cachexia, regardless of the model, needs to be addressed. With the help of next-generation sequencing, we are able to understand the profile of atrophied muscles at the transcriptional level.

Accordingly, 6 high-throughput transcriptome datasets, including one generated by us, were retrieved and analyzed. A preliminary study of the DEGs of cachexia and normal muscle tissues of each dataset showed an overlap of upregulated 43 genes in all the models (Fig. S7). However, no biological pathways were significantly enriched among them, possibly because of the small number of genes or because the underlying mechanism might be accomplished by different genes at the biological level. Therefore, we tried to integrate all datasets as one large high-throughput result and compared the profile between all atrophic and normal samples.

As the data were obtained from different platforms, the transcripts of each dataset varied significantly, and strong batch effects were observed. Batch effects are subgroups of measurements that behave differently across conditions but are unrelated to the biological variables in a study [37]. Although they are difficult or impossible to detect in low-dimensional assays such as qPCR or northern blot assays, high-throughput technologies provide enough data to detect and possibly remove these batch effects. In gene expression studies, the greatest source of differential expression is nearly always across batches rather than across biological groups, owing to the influence of technical artifacts [13]. Thus, the removal of batch effects and the preservation of biological information after this process were critical. In our study, the ComBat function based on the surrogate variable analysis (SVA) algorithm, which adopts parametric empirical Bayesian adjustments of known phenotypes, was applied to the data, and the adjusted data showed minimum existence of the batch effect. The samples were separated according to cachectic conditions.

The comparison of the results of the C26 model with the merged data indicated that there was more overlap of the data at the pathway level than at the gene level, suggesting that different genes in different models might accomplish the same biological functions. Notably, the JAK-STAT pathway was enriched in the merged data but not in the C26 model. However, previous works by our group and others have indicated that the STAT3 pathway is important in cancer cachexia in the muscle. Indeed, manual screening of the genes in the JAK-STAT pathway (mmu04630) showed that multiple genes, such as Stat1, Pias4, Stat5b, and Stat3 were significantly differentially expressed in C26 model muscles compared with control mouse muscles (Fig. S8A and B). In the C26 model, these genes were significantly altered, although they did not vary strongly enough to meet the DEG cutoff. Furthermore, validation at the protein level showed that Stat3 was markedly activated in the C26 muscle, consistent with previous findings (Fig. S8C). These findings also reminded us of the importance of the biological validation of high-throughput assay results.

Construction of the protein–protein interaction network by the enriched genes revealed one key gene, “Ddit4”, also known as “Redd1”, that was also significantly differentially expressed in all datasets (Fig. 6A and Fig. S4), which drew our attention. Ddit4 is a cytoplasmic protein originally characterized by its transcriptional upregulation in the setting of DNA damage. Its expression is induced by multiple cellular stresses, such as hypoxia [25, 38], heat shock [39], and energy depletion [40]. Interestingly, it was reported that Ddit4 inhibited protein synthesis in mouse skeletal muscle by inhibiting mTORC1 in fasting conditions [41]. Thus, we tried to explore the possible contribution of Ddit4 to muscle atrophy in other models, such as denervation, immobilization, et al. Unlike cancer cachexia models, the muscle in these models showed a more heterogeneous feature; there were no DEGs, including Ddit4 or pathways shared in all these models. However, at the pathway level, downstream pathways in muscle atrophy such as autophagy that accomplished the degradation of proteins were shared in multiple models, suggesting that different factors may stimulate these effects. Ddit4 might be critical in cancer cachexia which showed a unique response to metabolic stress in the muscle, causing continued degradation of organelles and proteins.

Recent works have shown that both inflammatory factors and fatty acid oxidation could induce muscle atrophy in a p38 dependent manner [42]. However, how p38 induced autophagy and/or protein degradation was not fully defined. In our study, p38 induced Ddit4 expression and transcription, which inhibited mTOR activity and induced autophagy in myocyte upon stimulation.

However, an interesting paradox was observed in cachexia mouse models. The transcriptional levels of genes of important members of the PI3K-AKT pathway, such as Pik3r1, Pik3r2, Akt1, Akt2, and Pdpk1 were all significantly upregulated in atrophied muscles, while the phosphorylated form of Akt1 was inhibited in the C26 model. This phenomenon might be explained by an autonomous rebounding reaction of muscle cells after sensing insufficient PI3K-AKT pathway activity. Although the cells tried to compensate for the inhibition of the pathway by upregulation of the transcription of the genes, the posttranscriptional regulation still blocked the function of the pathway and induced autophagy and atrophy of the muscle in cancer cachexia. This finding also reminds us that a study of the proteomic profile of atrophied muscles may reflect the pathology of the disease better than a study of the transcriptomic profile.

In addition, the limitations of our studies should also be addressed. First, only genes shared by all datasets were reserved for further integration. Thus, it was possible that some biologically meaningful transcripts were excluded from further analysis. In addition, the sample size of each model was not even; thus, models with a larger number of samples have greater weight in the merged data. It might be fairer to randomly sample the data to obtain an equivalent sample size for each model. Second, the transcripts were mapped to genes that were already annotated, and only the level of the highest transcript variant of the same gene was chosen for further investigation. Information on de novo transcript or transcript variants was not included in our study. Third, although we were focusing on the commonalities of the muscle transcript profiles in cancer cachexia, the specificity of each model should not be neglected. A further systematic comparison of the significance of each model and the clinical relevance should also be thoroughly investigated in the future.

## Supplementary information

Supplementary Figures and Tables
