# Supplementary material for: An integrative transcriptome study reveals Ddit4/Redd1 as a key regulator of cancer cachexia in rodent models
Source: Cell Death Dis. 2021 Jun 26;12(7):652. doi: 10.1038/s41419-021-03932-0 (PMC8236061; doi:10.1038/s41419-021-03932-0)
Supplement: Supplementary file 1 — Supplementary Figures and Tables [file 41419_2021_3932_MOESM1_ESM.pdf]

Supplementary Figures and Tables

Supplementary Figure S1

A

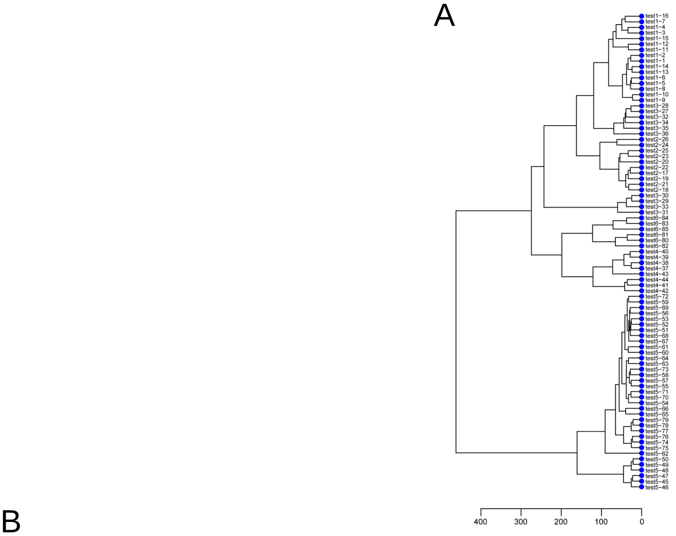

B

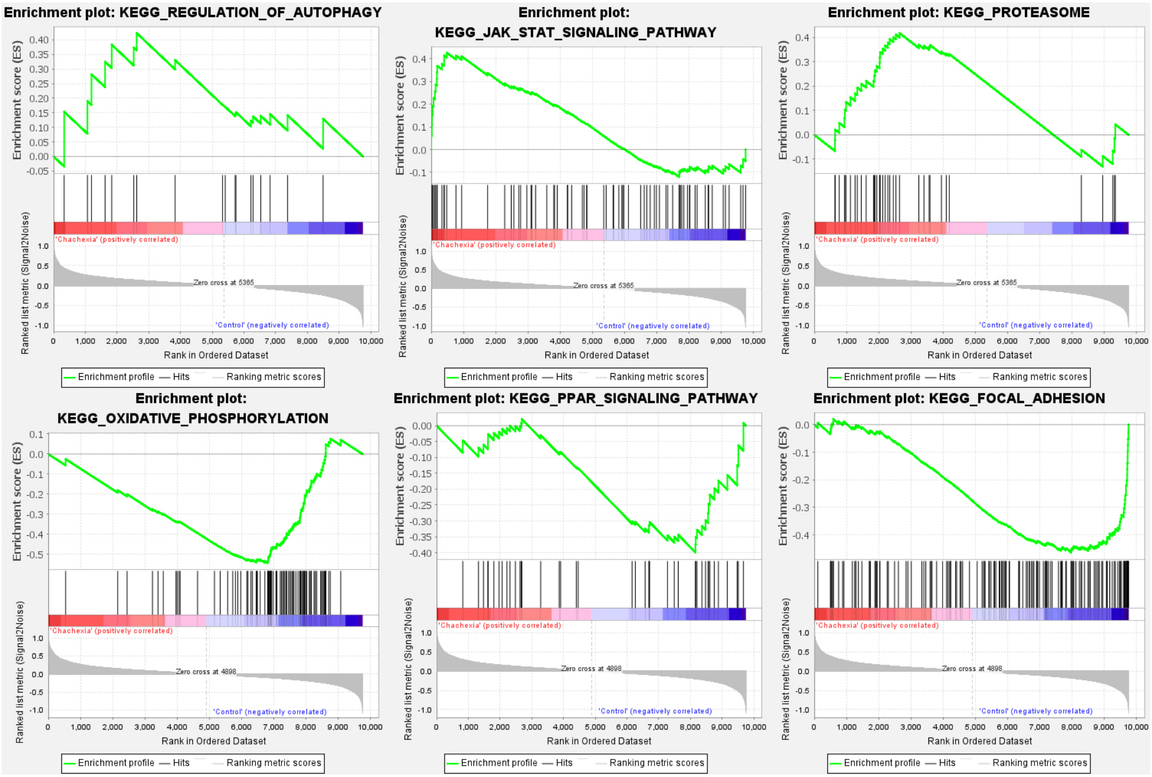

(A) Hierarchical Clustering assay of the transcriptome of the merged data and the samples were grouped according the data source.

(B) GSEA analysis of the human homolog of the genes

Supplementary Figure S2

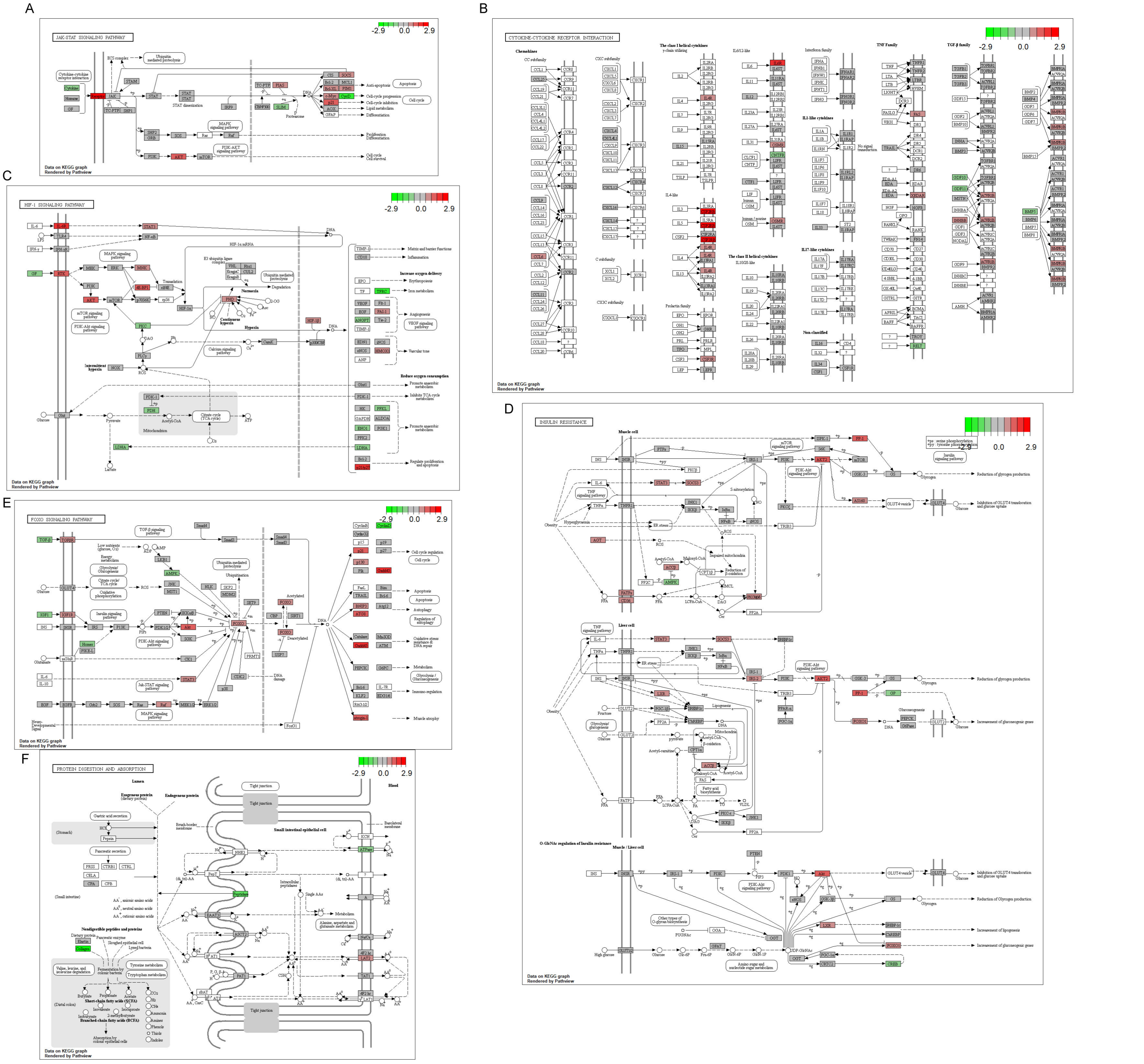

The DEGs were mapped to corresponding KEGG pathways, the colour represented the fold change of the genes. Red upregulated, green downregulated.

(A) JAK-STAT signalling pathway (mmu04630)

(B) Cytokine-cytokine receptor interaction (mmu04060)

(C) HIF-1 signalling pathway (mmu04066)

(D) Insulin resistance (mmu04931)

(E) FoxO signalling pathway (mmu04068)

(F) Protein digestion and absorption (mmu04974).

Supplementary Figure S3

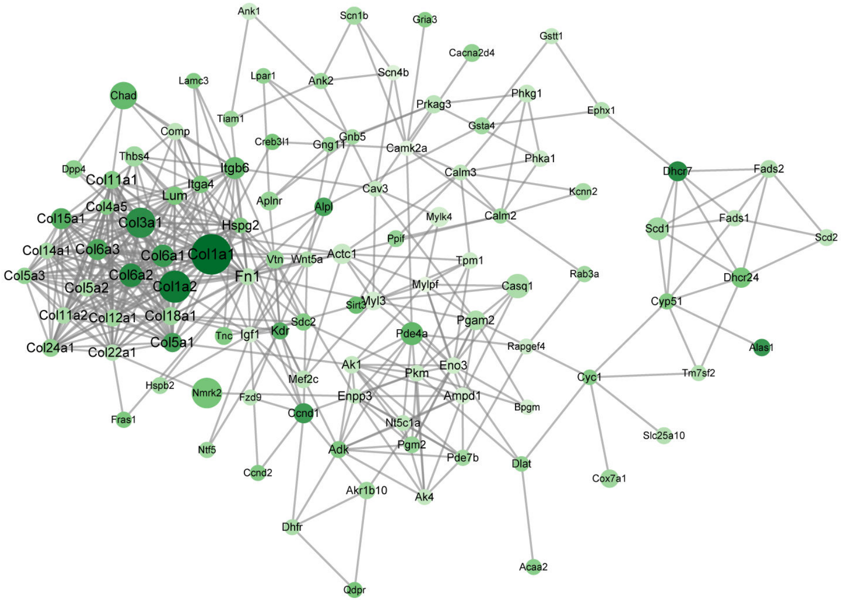

The network was constructed according their biological association. The colour of the node was mapped to the degree of the gene and the circle and font size of the node was mapped to the Number of direct edges.

Supplementary Figure S4

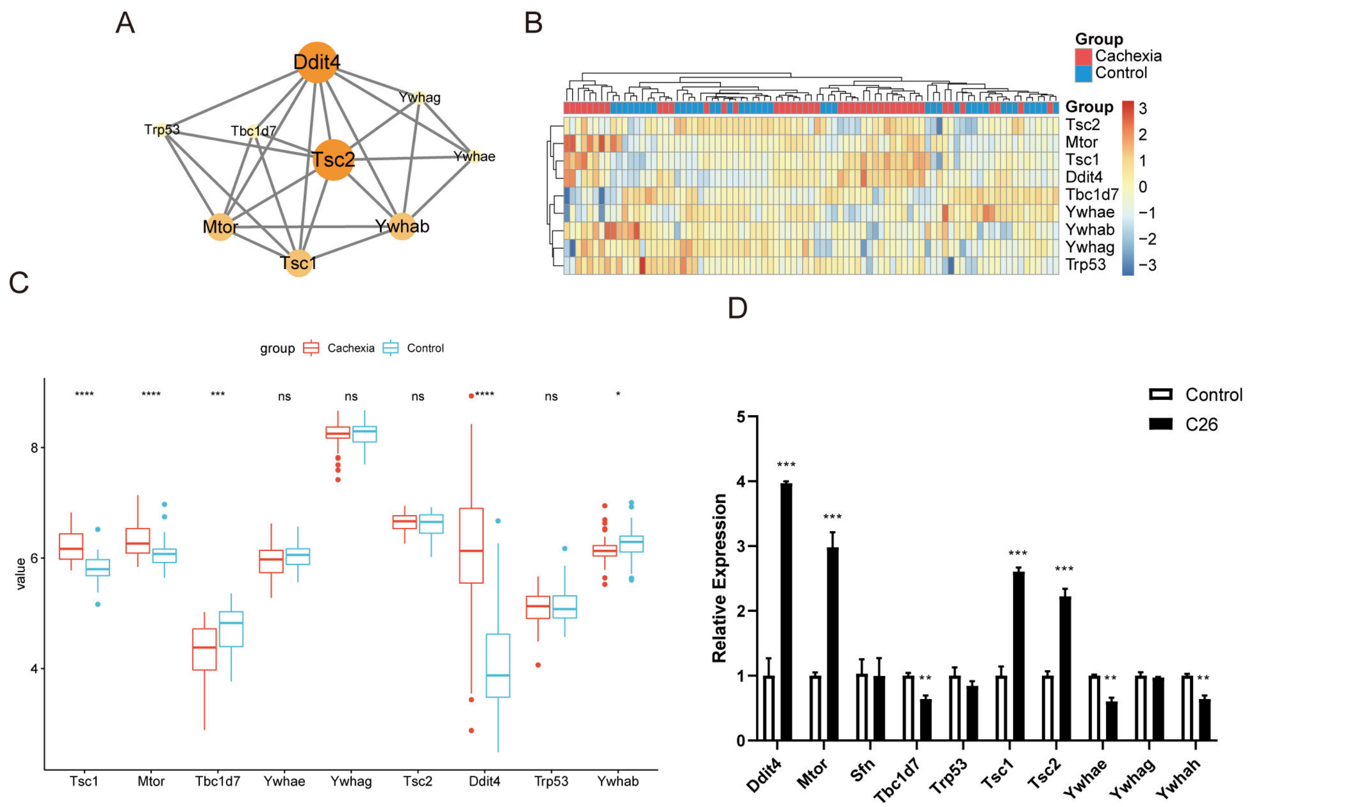

(A) Protein-protein interaction network of Ddit4 related genes. The colour of the node was mapped to the degree of the gene and the circle and font size of the node was mapped to the Number of direct edges.
(B) Heatmap of Ddit4 related genes of merged data.
(C) Boxplot of Ddit4 related genes at transcription level of merged data.
(D) qPCR validation of Ddit4 and mTOR related genes at transcriptional level in C26 cachexia models. Data were expressed as mean ± SD, *P < 0.05, **P < 0.01, ***P < 0.001, ****P < 0.0001. N=10

Supplementary Figure S5

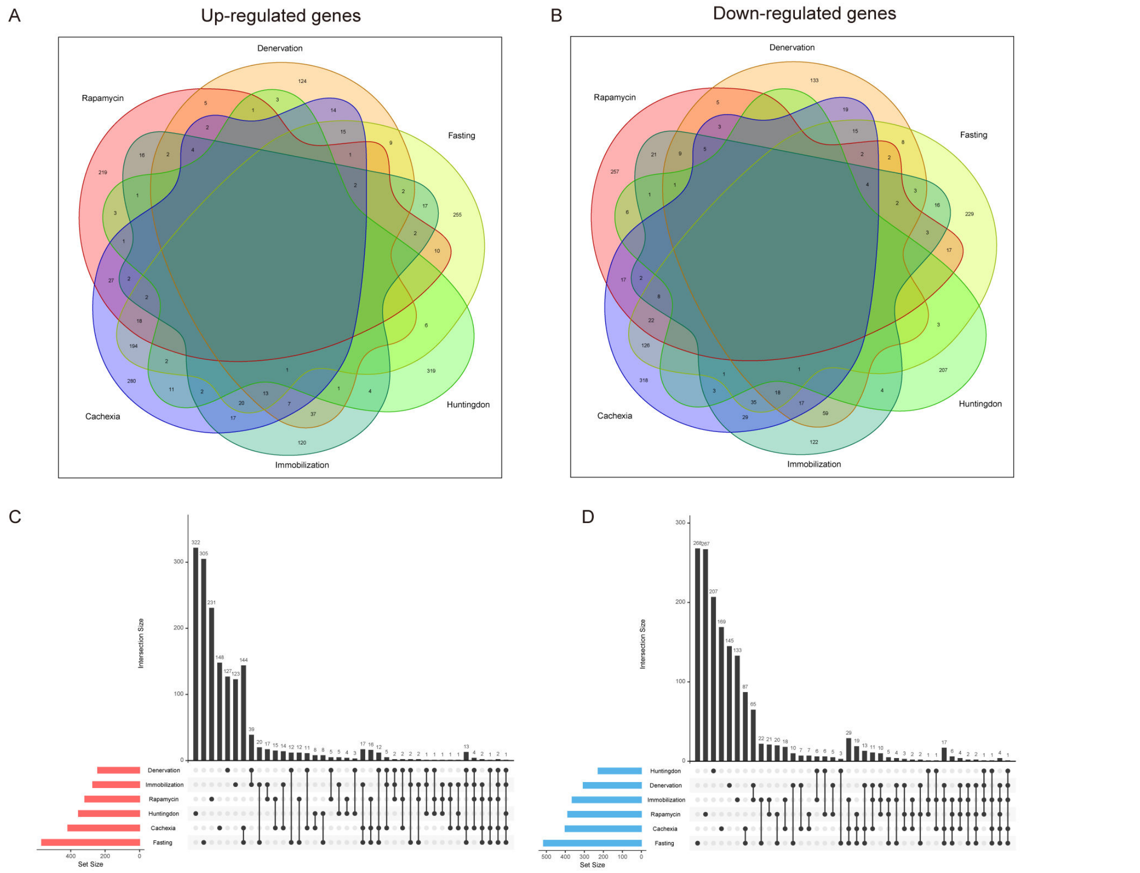

The Venn diagram showed the overlapping of the numbers of upregulated (A) and downregulated (B) DEGs discovered in each dataset, respectively. Same data was presented by upset figures in (C) and (D) for a clear view.

Supplementary Figure S6

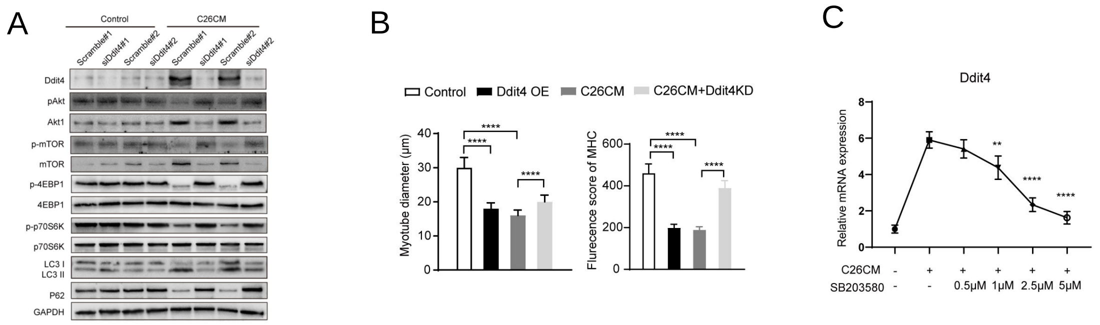

(A)Western blot ananlysis of indicated genes in C2C12 treated with RPMI1640 and C26CM with or without two different Ddit4siRNAs.

(B)The quantification of the average myotube diameter and the fluorescence intensity of MHC of myotubes.

(C) qPCR analysis of Ddit4 in C2C12 myotubes treated with C26 CM and SB203580.

Data are expressed as the mean ± SD, *P < 0.05, **P < 0.01, ***P < 0.001, ****P < 0.0001.

Supplementary Figure S7

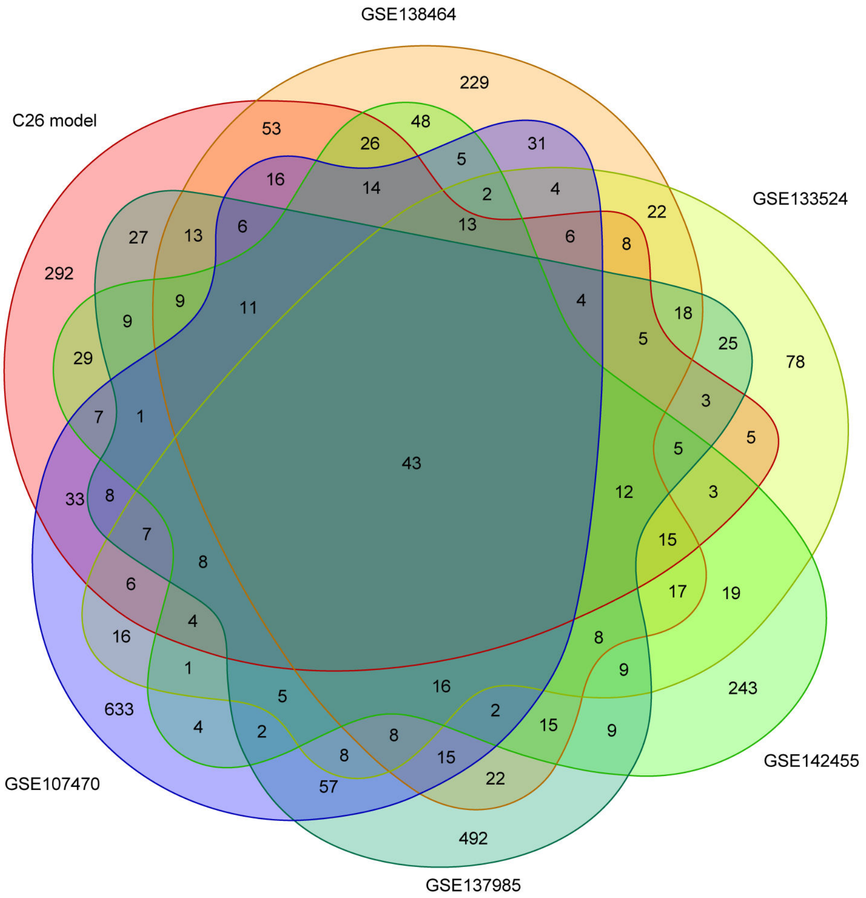

The Venn diagram showed the overlapping of the numbers of up-regulated 43 genes in all the models.

Supplementary Figure S8

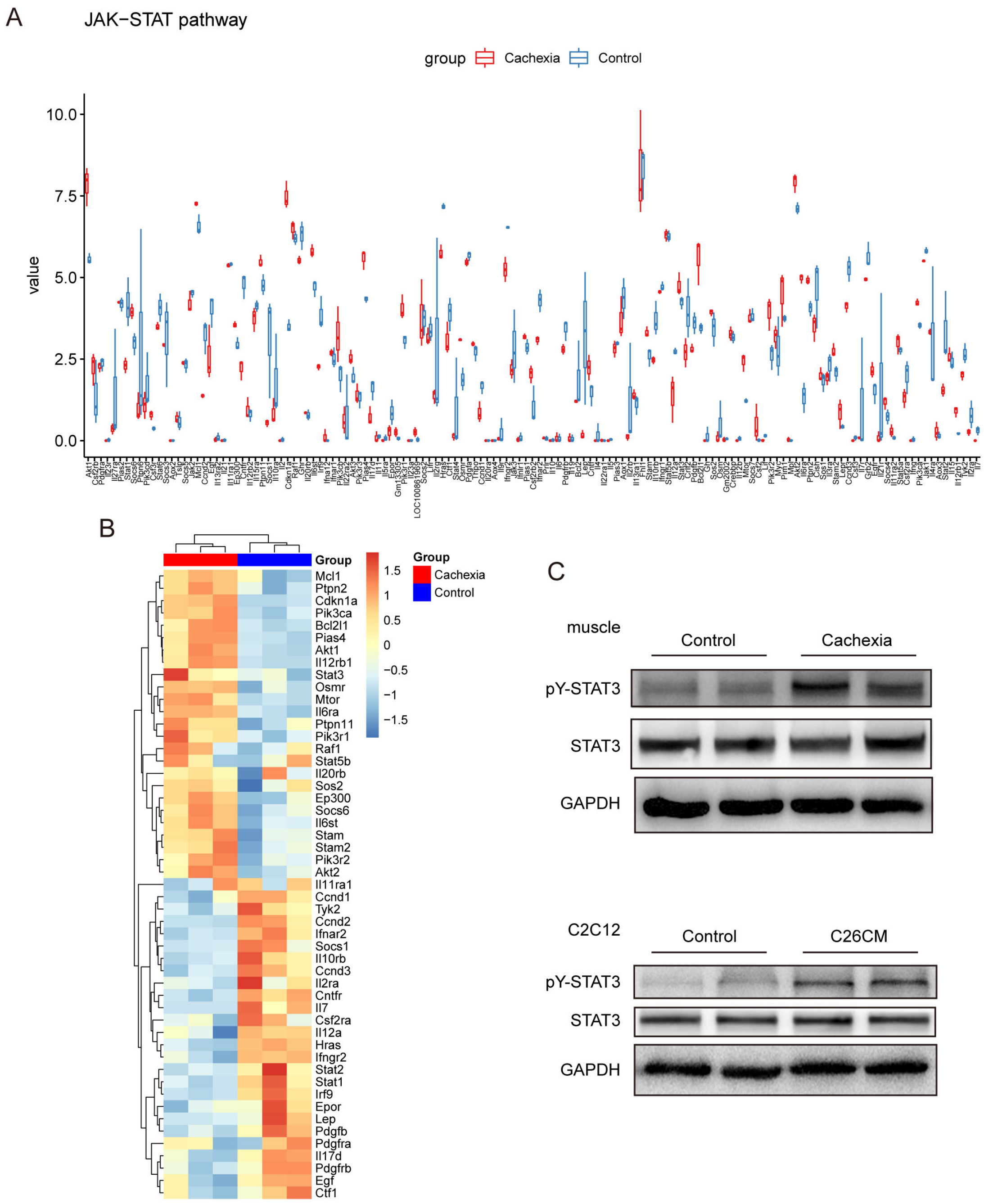

(A) Boxplot of the transcription level of genes of JAK-STAT pathway (mmu04630) in cachectic and control muscle of C26 model.
(B) Heatmap of JAK-STAT pathway related genes in C26 and normal mice muscle.
(C) Phosphorylation of STAT3 in C26 cachectic mice muscle and C2C12 treated with C26 CM were validated by western blot.

Table S1-Patients info

General characteristics of patients	Normal	Cancer cachexia
Gender(female/male)	1/3	2/3
Age(years)	56.5 ± 3.28, n=4	61.2 ± 3.22, n=5
Pre-illness weight (kg)	68 ± 5.24, n=4	69.6 ± 3.18, n=5
Current body mass (kg)	68 ± 5.07, n=4	60.8 ± 2.92, n=5
weight loss(%)	0	12.66 ± 0.83, n=5
BMI (kg/m2)	24.7 ± 0.9, n=4	24.5 ± 0.26, n=5
Malignancy	N/A	Hepatocellular Carcinoma

Table S2-Ddit4 siRNA

siDdit4#1: 5'- AGAGGAGGACGAGAAACGAUC-3';

siRNA scrambled#1: 5'- AAAGUACAGGCGAAAGAGGGC-3';

siDdit4#2 :5'-ACUUAACAGCCCCUGGAUCUU-3';

siRNA scrambled#2: 5'-UAAACUAACGGGUCUCCUCUC-3'.

Table S2-Primer list

Target	Sequence
Bnip3	Forward: 5‘-TACCTCTCGGTGACCGCC-3‘<br>Reverse: 5‘-AGTGCAGTTCTACCCAGGAG-3‘
Akt1	Forward: 5‘-AGAAGAGACGATGGACTTCCG-3‘<br>Reverse: 5‘-TCAAACTCGTTCATGGTCACAC-3‘
Foxo1	Forward: 5‘-ACGAGTGGATGGTGAAGAGC-3‘<br>Reverse: 5‘-AAGGGACAGATTGTGGCGAA-3‘
Irs2	Forward: 5‘-CCGAGTCAATAGCGGAGACC-3‘<br>Reverse: 5‘-GCTCAGGGGTCTATCCATGC-3‘
Eif4ebp1	Forward: 5‘-AGCAGCCCGGAAGATAAGC-3‘<br>Reverse: 5‘-ATCATTGCGTCCTACGGCTG-3‘
Il6ra	Forward: 5‘-CACGGGCACTCCTTGGATAG-3‘<br>Reverse: 5‘-CAACAGAGACCTGTGTGGGG-3‘
Ctsl	Forward: 5‘-CTGTTGCTATGGACGCAAGC-3‘<br>Reverse: 5‘-CCATGGTCGAGGTTCTTGCT-3‘
Egln3g	Forward: 5‘-ATGGTGATGGCCGCTGTATC-3‘<br>Reverse: 5‘-TCCGGAAATATCCGCAGGAC-3‘
Mknk2	Forward: 5‘-AGAACAGCCGAACTTCAGGG-3‘<br>Reverse: 5‘-GTGGGCTGAAGTCAGAGTCC-3‘
Cdkn1a	Forward: 5‘-TAAGGACGTCCCACTTTGCC-3‘<br>Reverse: 5‘-GACAACGGCACACTTTGCTC-3‘
Plcd4	Forward: 5‘-CTAGTGGGGGACCAGCTTTG-3‘<br>Reverse: 5‘-AGGCTCTCCATCAGGTCCAT-3‘
Phka1	Forward: 5‘-GGCAACTGGATGGACAGCTA-3‘<br>Reverse: 5‘-AACAGTGTGTGCCAAAAGGC-3‘
Pgam2g	Forward: 5‘-GCTGGCCAGAGAGTGCTTAT-3‘<br>Reverse: 5‘-GATTCCAGTGGGCAGGTTCA-3‘
Col1a1	Forward: 5‘-GACGCATGGCCAAGAAGACA-3‘<br>Reverse: 5‘-CTCGGGTTTCCACGTCTCAC-3‘
Camk2a	Forward: 5‘-ATTAGTCCGAGTGGAGCGAG-3‘<br>Reverse: 5‘-CCATCCCCGTCCTTGTCAAA-3‘
Calm2	Forward: 5‘-ATTAGTCCGAGTGGAGCGAG-3‘<br>Reverse: 5‘-CCATCCCCGTCCTTGTCAAA-3‘
Atp1b2	Forward: 5‘-AAGGAGTTCGTGTGGAACCC-3‘<br>Reverse: 5‘-TGAACATGGCCGTGAGGAAA-3‘
Bpgm	Forward: 5‘-GGCTGCTACTGGTAGTTTCCTT-3‘<br>Reverse: 5‘-ACATGCTGGTGGCTGAACTA-3‘
Pgm2	Forward: 5‘-GGCTGCTACTGGTAGTTTCCTT-3‘<br>Reverse: 5‘-ACATGCTGGTGGCTGAACTA-3‘
Scd1	Forward: 5‘-GGCCTGTACGGGATCATACTG-3‘<br>Reverse: 5‘-CCAGAGCGCTGGTCATGTAG-3‘
Ddit4	Forward: 5‘-GCCGGAGGAAGACTCCTCATA-3‘<br>Reverse: 5‘-CATCAGGTTGGCACACAGGT-3‘
mTOR	Forward: 5‘-GGGCAGTGTTGGCTCTACAT-3‘<br>Reverse: 5‘-CATAGGCTCGGCTGTAGCTC-3‘
TSC1	Forward: 5‘-TGATAGAGCAGGGAGCAGGT-3‘<br>Reverse: 5‘-GAGAGCCTCCAAAGTGGGTC-3‘
Tbc1d7	Forward: 5‘-CCTCCCGGATCTCTGAGTCT-3‘<br>Reverse: 5‘-TCCTCTGAGAGTCGTCAGTC-3‘
Ywhah	Forward: 5‘-CTACGACGATATGGCCTCCG-3‘<br>Reverse: 5‘-GCCTGGCACCAACTACATTC-3‘
Ywhae	Forward: 5‘-CTACGACGATATGGCCTCCG-3‘<br>Reverse: 5‘-GCCTGGCACCAACTACATTC-3‘
Ywhag	Forward: 5‘-TGTGAAGATGGTGGACCGC-3‘<br>Reverse: 5‘-ACAGTGGTTCGTTCAGCTCG-3‘
TSC2	Forward: 5‘-CACGGCCCTGTACAAGTCAT-3‘<br>Reverse: 5‘-CTCCTGGACTTCCCTCCTCA-3‘
Sfn	Forward: 5‘-ACAGGCCGAACGGTATGAAG-3‘

	Reverse: 5‘-GGAAAGCAGGTTTCGCTCCT-3‘
Trp53	Forward: 5‘-ATTCAGGCCCTCATCCTCCT-3‘
	Reverse: 5‘-TCCGACTGTGACTCCTCCAT-3‘
GAPDH	Forward: 5‘-GGAGAGTGTTTCCTCGTCCC-3‘
	Reverse: 5‘-AATCTCCACTTTGCCACTGC-3‘

Table S3-Go

	ID	Description	GeneRatio	BgRatio	pvalue	p.adjust	qvalue	geneID	Count
GO:0030198	GO:0030198	extracellular matrix orga	44/765	271/23210	1.69E-18	1.06E-14	7.80E-15	Tmem38b/Aebp1/Lgals3/Cyp1b1/A[illegible]	44
GO:0043062	GO:0043062	extracellular structure or	47/765	315/23210	3.77E-18	1.18E-14	8.71E-15	Tmem38b/Aebp1/Lgals3/Cyp1b1/C[illegible]	47
GO:0003012	GO:0003012	muscle system process	50/765	392/23210	2.44E-16	5.08E-13	3.75E-13	Fbxo32/Trim63/Scn3b/Gaa/Tmem3[illegible]	50
GO:0042692	GO:0042692	muscle cell differentiatio	46/765	395/23210	1.17E-13	1.83E-10	1.35E-10	Rora/Mecp2/Sik1/Dmd/Akt1/Myo1[illegible]	46
GO:0060537	GO:0060537	muscle tissue developm[illegible]	50/765	472/23210	3.89E-13	4.87E-10	3.59E-10	Sik1/Dmd/Myo18b/Alpk2/Myc/Maff	50
GO:0030199	GO:0030199	collagen fibril organizati	17/765	54/23210	8.08E-13	7.44E-10	5.50E-10	Aebp1/Cyp1b1/Lox/Col1a1/Col1a2/	17
GO:0014706	GO:0014706	striated muscle tissue de	48/765	449/23210	8.33E-13	7.44E-10	5.50E-10	Sik1/Dmd/Myo18b/Alpk2/Myc/Maff	48
GO:0031099	GO:0031099	regeneration	26/765	145/23210	1.70E-12	1.33E-09	9.81E-10	Igf1r/Cdkn1a/Hmox1/Cebpb/Fas/M[illegible]	26
GO:0051146	GO:0051146	striated muscle cell diffe	38/765	316/23210	6.25E-12	4.34E-09	3.21E-09	Sik1/Dmd/Akt1/Myo18b/Alpk2/Il4ra	38
GO:0010959	GO:0010959	regulation of metal ion t	44/765	421/23210	1.63E-11	1.02E-08	7.51E-09	Scn3b/Tmem38b/Wnk2/Ank3/Lgals[illegible]	44
GO:0007517	GO:0007517	muscle organ developm	44/765	424/23210	2.06E-11	1.17E-08	8.65E-09	Foxk1/Dmd/Myc/Maff/Des/Mylk2/M[illegible]	44
GO:0055001	GO:0055001	muscle cell developmen	29/765	207/23210	5.11E-11	2.49E-08	1.84E-08	Dmd/Myo18b/Alpk2/Myog/Naca/M	29
GO:0031589	GO:0031589	cell-substrate adhesion	38/765	339/23210	5.19E-11	2.49E-08	1.84E-08	Vwf/Acer2/Fam107a/Dmd/Pik3r1/S[illegible]	38
GO:0045785	GO:0045785	positive regulation of ce	42/765	411/23210	9.65E-11	4.31E-08	3.18E-08	Malt1/Selp/Il6ra/Tgm2/Ank3/Zbtb1[illegible]	42
GO:0055002	GO:0055002	striated muscle cell deve	27/765	193/23210	2.44E-10	1.02E-07	7.52E-08	Dmd/Myo18b/Alpk2/Myog/Naca/M	27
GO:0051188	GO:0051188	cofactor biosynthetic pr[illegible]	31/765	251/23210	2.85E-10	1.12E-07	8.24E-08	Foxk1/Ddit4/Sptb/Idh2/Acacb/Ier3/[illegible]	31
GO:0006091	GO:0006091	generation of precursor	39/765	379/23210	3.71E-10	1.37E-07	1.01E-07	Foxk1/Ddit4/Gaa/Mecp2/Bnip3/Idh[illegible]	39
GO:0009165	GO:0009165	nucleotide biosynthetic [illegible]	33/765	285/23210	4.19E-10	1.45E-07	1.07E-07	Foxk1/Ddit4/Entpd1/Idh2/Adcy2/Ac	33
GO:0009259	GO:0009259	ribonucleotide metaboli[illegible]	42/765	433/23210	4.87E-10	1.60E-07	1.18E-07	Foxk1/Ddit4/Entpd1/Rora/Mecp2/A[illegible]	42
GO:0072521	GO:0072521	purine-containing comp	45/765	490/23210	6.78E-10	2.12E-07	1.56E-07	Foxk1/Gda/Ddit4/Entpd1/Rora/Mec	45
GO:1901293	GO:1901293	nucleoside phosphate b	33/765	291/23210	7.20E-10	2.14E-07	1.58E-07	Foxk1/Ddit4/Entpd1/Idh2/Adcy2/Ac	33
GO:0009150	GO:0009150	purine ribonucleotide m	41/765	423/23210	8.03E-10	2.28E-07	1.69E-07	Foxk1/Ddit4/Entpd1/Rora/Mecp2/A[illegible]	41
GO:0006163	GO:0006163	purine nucleotide metab	42/765	447/23210	1.28E-09	3.48E-07	2.57E-07	Foxk1/Ddit4/Entpd1/Rora/Mecp2/A[illegible]	42
GO:0009108	GO:0009108	coenzyme biosynthetic [illegible]	26/765	194/23210	1.35E-09	3.51E-07	2.59E-07	Foxk1/Ddit4/Idh2/Acacb/Ier3/Pdk4/	26
GO:0006732	GO:0006732	coenzyme metabolic pr[illegible]	35/765	331/23210	1.45E-09	3.62E-07	2.68E-07	Foxk1/Ddit4/Fmo2/Idh2/Acacb/Ier3/	35
GO:0044262	GO:0044262	cellular carbohydrate m[illegible]	32/765	284/23210	1.53E-09	3.62E-07	2.68E-07	Foxk1/Rorc/Ddit4/Rora/Gaa/Mecp2/	32
GO:0019693	GO:0019693	ribose phosphate metab	42/765	450/23210	1.56E-09	3.62E-07	2.68E-07	Foxk1/Ddit4/Entpd1/Rora/Mecp2/A[illegible]	42
GO:0006941	GO:0006941	striated muscle contract[illegible]	23/765	158/23210	2.37E-09	5.30E-07	3.91E-07	Scn3b/Gaa/Tmem38b/Dmd/Kcna5/[illegible]	23
GO:0043502	GO:0043502	regulation of muscle ad[illegible]	18/765	97/23210	2.53E-09	5.46E-07	4.03E-07	Fbxo32/Trim63/Ece1/Foxo1/Foxo3/[illegible]	18
GO:0009132	GO:0009132	nucleoside diphosphate	20/765	121/23210	2.77E-09	5.76E-07	4.26E-07	Foxk1/Ddit4/Entpd1/Ier3/Stat3/Myc	20
GO:0009611	GO:0009611	response to wounding	44/765	495/23210	2.88E-09	5.81E-07	4.29E-07	Igf1r/Selp/Entpd1/Vwf/S100a9/Cdk[illegible]	44
GO:0009260	GO:0009260	ribonucleotide biosynth[illegible]	28/765	232/23210	3.50E-09	6.84E-07	5.05E-07	Foxk1/Ddit4/Entpd1/Adcy2/Acacb/I[illegible]	28
GO:0001666	GO:0001666	response to hypoxia	26/765	204/23210	3.99E-09	7.56E-07	5.58E-07	Eif4ebp1/Ddit4/Rora/Arnt/Mecp2/B[illegible]	26
GO:0009141	GO:0009141	nucleoside triphosphate	32/765	298/23210	5.04E-09	9.28E-07	6.85E-07	Foxk1/Ddit4/Entpd1/Mecp2/Ier3/Bc	32
GO:0009152	GO:0009152	purine ribonucleotide bi	27/765	223/23210	6.21E-09	1.11E-06	8.19E-07	Foxk1/Ddit4/Entpd1/Adcy2/Acacb/I[illegible]	27
GO:0046390	GO:0046390	ribose phosphate biosyr	28/765	239/23210	6.82E-09	1.15E-06	8.51E-07	Foxk1/Ddit4/Entpd1/Adcy2/Acacb/I[illegible]	28
GO:0090257	GO:0090257	regulation of muscle sys	28/765	239/23210	6.82E-09	1.15E-06	8.51E-07	Fbxo32/Trim63/Tmem38b/Dmd/Ece	28
GO:0006164	GO:0006164	purine nucleotide biosyr	28/765	240/23210	7.48E-09	1.23E-06	9.09E-07	Foxk1/Ddit4/Entpd1/Adcy2/Acacb/I[illegible]	28
GO:0001667	GO:0001667	ameboidal-type cell mig	38/765	406/23210	8.77E-09	1.40E-06	1.04E-06	Ptp4a3/Sema6b/Sema7a/Glul/Mecp	38
GO:0006936	GO:0006936	muscle contraction	31/765	289/23210	8.99E-09	1.40E-06	1.04E-06	Trim63/Scn3b/Gaa/Tmem38b/Dmd/	31
GO:0043500	GO:0043500	muscle adaptation	19/765	117/23210	9.30E-09	1.42E-06	1.05E-06	Fbxo32/Trim63/Klf15/Ece1/Foxo1/F[illegible]	19
GO:0072522	GO:0072522	purine-containing comp	28/765	245/23210	1.18E-08	1.76E-06	1.30E-06	Foxk1/Ddit4/Entpd1/Adcy2/Acacb/I[illegible]	28
GO:0042060	GO:0042060	wound healing	35/765	360/23210	1.30E-08	1.89E-06	1.39E-06	Selp/Entpd1/Vwf/S100a9/Cdkn1a/H	35
GO:0009142	GO:0009142	nucleoside triphosphate	22/765	160/23210	1.54E-08	2.19E-06	1.62E-06	Foxk1/Ddit4/Entpd1/Ier3/Bcl2l1/Ctp	22
GO:0007160	GO:0007160	cell-matrix adhesion	25/765	204/23210	1.77E-08	2.45E-06	1.81E-06	Acer2/Fam107a/Dmd/Pik3r1/Apod/	25
GO:0060538	GO:0060538	skeletal muscle organ d[illegible]	25/765	206/23210	2.15E-08	2.92E-06	2.16E-06	Dmd/Myc/Maff/Mylk2/Myog/Naca/[illegible]	25
GO:0009123	GO:0009123	nucleoside monophosph	30/765	285/23210	2.40E-08	3.12E-06	2.31E-06	Foxk1/Ddit4/Entpd1/Mecp2/Ier3/Bc	30
GO:0061448	GO:0061448	connective tissue develo	30/765	285/23210	2.40E-08	3.12E-06	2.31E-06	Rorc/Il6ra/Arid5a/Zbtb16/Arrdc3/M[illegible]	30
GO:0043270	GO:0043270	positive regulation of io[illegible]	32/765	322/23210	3.25E-08	4.15E-06	3.06E-06	Scn3b/Wnk2/Ank3/Lgals3/Dmd/Akt	32
GO:0009126	GO:0009126	purine nucleoside mono	29/765	273/23210	3.35E-08	4.19E-06	3.10E-06	Foxk1/Ddit4/Entpd1/Mecp2/Ier3/Bc	29
GO:1901342	GO:1901342	regulation of vasculatur[illegible]	33/765	341/23210	3.77E-08	4.50E-06	3.32E-06	Il6ra/Glul/Mecp2/Lgals3/Tmem100/[illegible]	33
GO:0009124	GO:0009124	nucleoside monophosph	22/765	168/23210	3.80E-08	4.50E-06	3.32E-06	Foxk1/Ddit4/Entpd1/Ier3/Bcl2l1/Stat	22
GO:0007519	GO:0007519	skeletal muscle tissue de	24/765	197/23210	3.81E-08	4.50E-06	3.32E-06	Dmd/Myc/Maff/Mylk2/Myog/Naca/[illegible]	24
GO:0008015	GO:0008015	blood circulation	41/765	484/23210	3.89E-08	4.50E-06	3.33E-06	Ptp4a3/Scn3b/Gaa/Tmem38b/Mecp	41
GO:0009199	GO:0009199	ribonucleoside triphosph	29/765	276/23210	4.27E-08	4.69E-06	3.47E-06	Foxk1/Ddit4/Entpd1/Mecp2/Ier3/Bc	29
GO:0006165	GO:0006165	nucleoside diphosphate	17/765	103/23210	4.35E-08	4.69E-06	3.47E-06	Foxk1/Ddit4/Ier3/Stat3/Myc/Myog/[illegible]	17
GO:0009135	GO:0009135	purine nucleoside dipho	17/765	103/23210	4.35E-08	4.69E-06	3.47E-06	Foxk1/Ddit4/Entpd1/Ier3/Stat3/Myc	17

GO:0009179	GO:0009179	purine ribonucleoside di	17/765	103/23210	4.35E-08	4.69E-06	3.47E-06	Foxk1/Ddit4/Entpd1/Ier3/Stat3/Myc	17
GO:0009636	GO:0009636	response to toxic substa	33/765	344/23210	4.66E-08	4.93E-06	3.64E-06	Igf1r/Lcn2/Sesn1/Mt2/Arnt/Mt1/Gst	33
GO:0036293	GO:0036293	response to decreased c	26/765	230/23210	4.89E-08	5.10E-06	3.76E-06	Eif4ebp1/Ddit4/Rora/Arnt/Mecp2/B	26
GO:0046034	GO:0046034	ATP metabolic process	27/765	247/23210	5.49E-08	5.62E-06	4.15E-06	Foxk1/Ddit4/Entpd1/Mecp2/Ier3/Bc	27
GO:0034404	GO:0034404	nucleobase-containing s	22/765	172/23210	5.83E-08	5.63E-06	4.16E-06	Foxk1/Ddit4/Entpd1/Ier3/Ctps2/Stat	22
GO:0014897	GO:0014897	striated muscle hypertro	17/765	105/23210	5.84E-08	5.63E-06	4.16E-06	Fbxo32/Trim63/Klf15/Ece1/Foxo1/E	17
GO:0046939	GO:0046939	nucleotide phosphorylat	17/765	105/23210	5.84E-08	5.63E-06	4.16E-06	Foxk1/Ddit4/Ier3/Stat3/Myc/Myog/S	17
GO:0009127	GO:0009127	purine nucleoside mono	21/765	158/23210	5.95E-08	5.63E-06	4.16E-06	Foxk1/Ddit4/Entpd1/Ier3/Bcl2l1/Stat	21
GO:0009168	GO:0009168	purine ribonucleoside m	21/765	158/23210	5.95E-08	5.63E-06	4.16E-06	Foxk1/Ddit4/Entpd1/Ier3/Bcl2l1/Stat	21
GO:0009144	GO:0009144	purine nucleoside triphc	29/765	281/23210	6.32E-08	5.90E-06	4.35E-06	Foxk1/Ddit4/Entpd1/Mecp2/Ier3/Bc	29
GO:1904062	GO:1904062	regulation of cation tran	33/765	349/23210	6.57E-08	6.03E-06	4.46E-06	Scn3b/Tmem38b/Wnk2/Ank3/Dmd/	33
GO:0040013	GO:0040013	negative regulation of lc	32/765	332/23210	6.66E-08	6.03E-06	4.46E-06	Sema6b/Sema7a/Mecp2/Idh2/Akt1/	32
GO:0051271	GO:0051271	negative regulation of ce	31/765	316/23210	7.16E-08	6.35E-06	4.69E-06	Sema6b/Sema7a/Mecp2/Idh2/Akt1/	31
GO:0003013	GO:0003013	circulatory system proce	41/765	495/23210	7.22E-08	6.35E-06	4.69E-06	Ptp4a3/Scn3b/Gaa/Tmem38b/Mecp	41
GO:0009185	GO:0009185	ribonucleoside diphosph	17/765	107/23210	7.78E-08	6.66E-06	4.92E-06	Foxk1/Ddit4/Entpd1/Ier3/Stat3/Myc	17
GO:0014896	GO:0014896	muscle hypertrophy	17/765	107/23210	7.78E-08	6.66E-06	4.92E-06	Fbxo32/Trim63/Klf15/Ece1/Foxo1/E	17
GO:0034765	GO:0034765	regulation of ion transm	40/765	478/23210	7.93E-08	6.69E-06	4.94E-06	Scn3b/Tmem38b/Wnk2/Ank3/Dmd/	40
GO:0009156	GO:0009156	ribonucleoside monoph	21/765	162/23210	9.21E-08	7.68E-06	5.67E-06	Foxk1/Ddit4/Entpd1/Ier3/Bcl2l1/Stat	21
GO:0045444	GO:0045444	fat cell differentiation	26/765	238/23210	9.77E-08	8.03E-06	5.93E-06	Cebpd/Rorc/Rora/Bnip3/Zbtb16/Gp	26
GO:0009167	GO:0009167	purine ribonucleoside m	28/765	272/23210	1.12E-07	9.00E-06	6.64E-06	Foxk1/Ddit4/Entpd1/Mecp2/Ier3/Bc	28
GO:0009205	GO:0009205	purine ribonucleoside tr	28/765	272/23210	1.12E-07	9.00E-06	6.64E-06	Foxk1/Ddit4/Entpd1/Mecp2/Ier3/Bc	28
GO:0014743	GO:0014743	regulation of muscle hyp	14/765	74/23210	1.16E-07	9.16E-06	6.77E-06	Fbxo32/Trim63/Ece1/Foxo1/Errfi1/A	14
GO:0045765	GO:0045765	regulation of angiogene	30/765	307/23210	1.26E-07	9.77E-06	7.22E-06	Glul/Mecp2/Lgals3/Hmox1/Stat3/Ru	30
GO:0046031	GO:0046031	ADP metabolic process	16/765	98/23210	1.27E-07	9.77E-06	7.22E-06	Foxk1/Ddit4/Ier3/Stat3/Myc/Ampd3	16
GO:0060048	GO:0060048	cardiac muscle contracti	18/765	124/23210	1.35E-07	1.03E-05	7.58E-06	Scn3b/Gaa/Tmem38b/Dmd/Kcna5/	18
GO:0010810	GO:0010810	regulation of cell-substr	24/765	211/23210	1.42E-07	1.06E-05	7.80E-06	Acer2/Fam107a/Dmd/Pik3r1/Apod/	24
GO:0051216	GO:0051216	cartilage development	24/765	211/23210	1.42E-07	1.06E-05	7.80E-06	Arid5a/Zbtb16/Mustn1/Bmpr1b/Col	24
GO:0009161	GO:0009161	ribonucleoside monoph	28/765	276/23210	1.52E-07	1.12E-05	8.28E-06	Foxk1/Ddit4/Entpd1/Mecp2/Ier3/Bc	28
GO:0048009	GO:0048009	insulin-like growth facto	10/765	36/23210	1.67E-07	1.21E-05	8.95E-06	Igf1r/Akt1/Pik3r1/Igfbp3/Trim72/Igf	10
GO:0015672	GO:0015672	monovalent inorganic ca	38/765	455/23210	1.76E-07	1.27E-05	9.35E-06	Slc12a2/Slc10a6/Scn3b/Tmem38b/W	38
GO:0010631	GO:0010631	epithelial cell migration	28/765	280/23210	2.06E-07	1.44E-05	1.07E-05	Ptp4a3/Glul/Mecp2/Akt1/Hmox1/Cy	28
GO:0060348	GO:0060348	bone development	25/765	231/23210	2.06E-07	1.44E-05	1.07E-05	Tmem38b/Spns2/Dym/Lox/Ryr1/Co	25
GO:0005977	GO:0005977	glycogen metabolic proc	14/765	78/23210	2.30E-07	1.56E-05	1.15E-05	Gaa/Akt1/Gyg/Ppp1r1a/Ppp1r3c/Gr	14
GO:0006073	GO:0006073	cellular glucan metaboli	14/765	78/23210	2.30E-07	1.56E-05	1.15E-05	Gaa/Akt1/Gyg/Ppp1r1a/Ppp1r3c/Gr	14
GO:0044042	GO:0044042	glucan metabolic proces	14/765	78/23210	2.30E-07	1.56E-05	1.15E-05	Gaa/Akt1/Gyg/Ppp1r1a/Ppp1r3c/Gr	14
GO:0070482	GO:0070482	response to oxygen leve	28/765	282/23210	2.38E-07	1.58E-05	1.17E-05	Eif4ebp1/Ddit4/Rora/Arnt/Mecp2/B	28
GO:0090132	GO:0090132	epithelium migration	28/765	282/23210	2.38E-07	1.58E-05	1.17E-05	Ptp4a3/Glul/Mecp2/Akt1/Hmox1/Cy	28
GO:0002028	GO:0002028	regulation of sodium ior	15/765	91/23210	2.74E-07	1.79E-05	1.33E-05	Scn3b/Wnk2/Ank3/Sik1/Dmd/Akt1/	15
GO:0090130	GO:0090130	tissue migration	28/765	284/23210	2.76E-07	1.79E-05	1.33E-05	Ptp4a3/Glul/Mecp2/Akt1/Hmox1/Cy	28
GO:0006090	GO:0006090	pyruvate metabolic proc	17/765	118/23210	3.34E-07	2.15E-05	1.59E-05	Foxk1/Ddit4/Ier3/Pdk4/Stat3/Myc/M	17
GO:0019362	GO:0019362	pyridine nucleotide meta	20/765	160/23210	3.38E-07	2.16E-05	1.59E-05	Foxk1/Ddit4/Fmo2/Idh2/Ier3/Stat3/	20
GO:0042326	GO:0042326	negative regulation of p	37/765	452/23210	4.19E-07	2.62E-05	1.93E-05	Gadd45a/Igf1r/Ddit4/Wnk2/Cblb/Ra	37
GO:0006733	GO:0006733	oxidoreduction coenzym	21/765	177/23210	4.19E-07	2.62E-05	1.93E-05	Foxk1/Ddit4/Fmo2/Idh2/Ier3/Stat3/	21
GO:0042866	GO:0042866	pyruvate biosynthetic pr	15/765	94/23210	4.24E-07	2.62E-05	1.93E-05	Foxk1/Ddit4/Ier3/Stat3/Myc/Myog/S	15
GO:0002062	GO:0002062	chondrocyte differentiat	17/765	120/23210	4.27E-07	2.62E-05	1.93E-05	Arid5a/Zbtb16/Mustn1/Bmpr1b/Col	17
GO:0009145	GO:0009145	purine nucleoside triphc	19/765	148/23210	4.36E-07	2.62E-05	1.94E-05	Foxk1/Ddit4/Entpd1/Ier3/Bcl2l1/Stat	19
GO:1901292	GO:1901292	nucleoside phosphate ca	19/765	148/23210	4.36E-07	2.62E-05	1.94E-05	Foxk1/Ddit4/Entpd1/Ier3/Stat3/Myc	19
GO:0010611	GO:0010611	regulation of cardiac mu	13/765	71/23210	4.88E-07	2.89E-05	2.14E-05	Fbxo32/Trim63/Ece1/Foxo1/Errfi1/A	13
GO:0006006	GO:0006006	glucose metabolic proce	22/765	194/23210	4.92E-07	2.89E-05	2.14E-05	Foxk1/Rorc/Rora/Acacb/Sik1/Akt1/F	22
GO:0001503	GO:0001503	ossification	33/765	381/23210	5.01E-07	2.89E-05	2.14E-05	Cebpd/Tmem38b/Zbtb16/Akt1/Igfb	33
GO:0014874	GO:0014874	response to stimulus inv	6/765	11/23210	5.04E-07	2.89E-05	2.14E-05	Fbxo32/Trim63/Myog/Nol3/Agt/Prk	6
GO:0097421	GO:0097421	liver regeneration	6/765	11/23210	5.04E-07	2.89E-05	2.14E-05	Hmox1/Cebpb/Ezh1/Ccnd1/Vtn/Au	6
GO:0009201	GO:0009201	ribonucleoside triphosph	19/765	150/23210	5.38E-07	3.06E-05	2.26E-05	Foxk1/Ddit4/Entpd1/Ier3/Bcl2l1/Ctp	19
GO:0060047	GO:0060047	heart contraction	23/765	211/23210	5.52E-07	3.08E-05	2.28E-05	Scn3b/Gaa/Tmem38b/Sp4/Dmd/Pik	23
GO:0048738	GO:0048738	cardiac muscle tissue de	26/765	260/23210	5.56E-07	3.08E-05	2.28E-05	Sik1/Myo18b/Alpk2/Mylk2/Pim1/Nr	26
GO:0072524	GO:0072524	pyridine-containing com	20/765	165/23210	5.57E-07	3.08E-05	2.28E-05	Foxk1/Ddit4/Fmo2/Idh2/Ier3/Stat3/	20
GO:0019363	GO:0019363	pyridine nucleotide bios	16/765	109/23210	5.69E-07	3.12E-05	2.30E-05	Foxk1/Ddit4/Idh2/Ier3/Stat3/Myc/M	16
GO:0006754	GO:0006754	ATP biosynthetic proces	18/765	137/23210	6.18E-07	3.36E-05	2.48E-05	Foxk1/Ddit4/Entpd1/Ier3/Bcl2l1/Stat	18

GO:0043542	GO:0043542	endothelial cell migratio	22/765	198/23210	6.99E-07	3.76E-05	2.78E-05	Ptp4a3/Glul/Mecp2/Akt1/Hmox1/Cy	22
GO:0010721	GO:0010721	negative regulation of c	33/765	387/23210	7.13E-07	3.81E-05	2.81E-05	Dnm3/Sema6b/Sema7a/Mecp2/App	33
GO:0007162	GO:0007162	negative regulation of c	27/765	284/23210	9.18E-07	4.86E-05	3.59E-05	Acer2/Cblb/Lgals3/Fam107a/Akt1/P	27
GO:0072525	GO:0072525	pyridine-containing con	16/765	113/23210	9.36E-07	4.91E-05	3.63E-05	Foxk1/Ddit4/Idh2/Ier3/Stat3/Myc/M	16
GO:0071496	GO:0071496	cellular response to exte	27/765	285/23210	9.83E-07	5.12E-05	3.78E-05	Sesn1/Glul/Fam107a/Sik1/Map1lc3b	27
GO:0030336	GO:0030336	negative regulation of c	26/765	268/23210	9.93E-07	5.13E-05	3.79E-05	Mecp2/Idh2/Akt1/Igfbp3/Apod/Stat	26
GO:0006112	GO:0006112	energy reserve metaboli	14/765	88/23210	1.07E-06	5.42E-05	4.00E-05	Gaa/Akt1/Gyg/Ppp1r1a/Ppp1r3c/Gr	14
GO:0035914	GO:0035914	skeletal muscle cell diffe	14/765	88/23210	1.07E-06	5.42E-05	4.00E-05	Myc/Maff/Mylk2/Myog/Btg2/Rbfox1	14
GO:0003300	GO:0003300	cardiac muscle hypertro	15/765	101/23210	1.10E-06	5.53E-05	4.09E-05	Fbxo32/Trim63/Klf15/Ece1/Foxo1/Er	15
GO:0046496	GO:0046496	nicotinamide nucleotide	19/765	158/23210	1.20E-06	6.01E-05	4.44E-05	Foxk1/Ddit4/Fmo2/Idh2/Ier3/Stat3/I	19
GO:0015980	GO:0015980	energy derivation by oxi	25/765	254/23210	1.23E-06	6.08E-05	4.49E-05	Gaa/Mecp2/Bnip3/Idh2/Akt1/Myc/G	25
GO:0003015	GO:0003015	heart process	23/765	221/23210	1.24E-06	6.13E-05	4.53E-05	Scn3b/Gaa/Tmem38b/Sp4/Dmd/Pik	23
GO:0006096	GO:0006096	glycolytic process	14/765	90/23210	1.41E-06	6.89E-05	5.09E-05	Foxk1/Ddit4/Ier3/Stat3/Myc/Myog/S	14
GO:0031668	GO:0031668	cellular response to extr	23/765	223/23210	1.46E-06	7.05E-05	5.21E-05	Sesn1/Glul/Fam107a/Sik1/Map1lc3b	23
GO:0051147	GO:0051147	regulation of muscle cel	19/765	161/23210	1.60E-06	7.65E-05	5.65E-05	Mecp2/Sik1/Il4ra/Myog/Naca/Trim7	19
GO:0044264	GO:0044264	cellular polysaccharide n	15/765	104/23210	1.61E-06	7.65E-05	5.65E-05	Gaa/Akt1/Gyg/Ppp1r1a/B3gnt3/Ppp	15
GO:0006757	GO:0006757	ATP generation from AD	14/765	91/23210	1.62E-06	7.65E-05	5.65E-05	Foxk1/Ddit4/Ier3/Stat3/Myc/Myog/S	14
GO:0008016	GO:0008016	regulation of heart cont	20/765	177/23210	1.71E-06	8.02E-05	5.92E-05	Scn3b/Gaa/Tmem38b/Sp4/Dmd/Pik	20
GO:0009206	GO:0009206	purine ribonucleoside tr	18/765	147/23210	1.75E-06	8.18E-05	6.04E-05	Foxk1/Ddit4/Entpd1/Ier3/Bcl2l1/Stat	18
GO:0043266	GO:0043266	regulation of potassium	15/765	105/23210	1.82E-06	8.41E-05	6.21E-05	Wnk2/Ank3/Kcna5/Cd63/Abcc8/Aka	15
GO:0048588	GO:0048588	developmental cell grow	25/765	260/23210	1.88E-06	8.64E-05	6.38E-05	Sema6b/Sema7a/Mecp2/Impact/Tw	25
GO:0031669	GO:0031669	cellular response to nutr	21/765	194/23210	1.90E-06	8.69E-05	6.42E-05	Sesn1/Glul/Fam107a/Sik1/Map1lc3b	21
GO:0019359	GO:0019359	nicotinamide nucleotide	15/765	106/23210	2.05E-06	9.30E-05	6.87E-05	Foxk1/Ddit4/Idh2/Ier3/Stat3/Myc/M	15
GO:2000146	GO:2000146	negative regulation of c	26/765	281/23210	2.41E-06	0.000109	8.01E-05	Mecp2/Idh2/Akt1/Igfbp3/Apod/Stat	26
GO:0072330	GO:0072330	monocarboxylic acid bic	25/765	264/23210	2.48E-06	0.000111	8.17E-05	Foxk1/Ddit4/Acacb/Ier3/Pdk4/Acss1	25
GO:0042594	GO:0042594	response to starvation	19/765	166/23210	2.54E-06	0.000113	8.32E-05	Eif4ebp1/Foxk1/Sesn1/Glul/Sik1/Ma	19
GO:0048747	GO:0048747	muscle fiber developme	13/765	82/23210	2.68E-06	0.000118	8.72E-05	Dmd/Myo18b/Myog/Naca/Nrap/Lo:	13
GO:0051153	GO:0051153	regulation of striated mu	16/765	123/23210	2.94E-06	0.000129	9.50E-05	Sik1/Il4ra/Myog/Naca/Trim72/Tbx1/	16
GO:0034764	GO:0034764	positive regulation of tra	23/765	233/23210	3.09E-06	0.000134	9.90E-05	Wnk2/Ank3/Dmd/Akt1/Klf15/Abcc8	23
GO:0070296	GO:0070296	sarcoplasmic reticulum c	8/765	29/23210	3.12E-06	0.000134	9.91E-05	Tmem38b/Dmd/Cacng1/Nol3/Ryr1/	8
GO:0022407	GO:0022407	regulation of cell-cell ac	32/765	395/23210	3.13E-06	0.000134	9.91E-05	Malt1/Il6ra/Cblb/Ank3/Zbtb16/Lgals	32
GO:0014808	GO:0014808	release of sequestered c	7/765	21/23210	3.19E-06	0.000136	0.000100243	Tmem38b/Dmd/Nol3/Ryr1/Ank2/Ca	7
GO:0014902	GO:0014902	myotube differentiation	16/765	124/23210	3.28E-06	0.000138	0.000102166	Sik1/Dmd/Il4ra/Myog/Naca/Trim72/	16
GO:0010035	GO:0010035	response to inorganic su	37/765	493/23210	3.30E-06	0.000138	0.000102166	Lcn2/Mt2/Mt1/Mecp2/Bnip3/Ank3/I	37
GO:0062012	GO:0062012	regulation of small mole	30/765	359/23210	3.41E-06	0.000142	0.000104953	Foxk1/Rorc/Ddit4/Entpd1/Rora/App	30
GO:1901379	GO:1901379	regulation of potassium	13/765	84/23210	3.54E-06	0.000146	0.000108136	Wnk2/Ank3/Cd63/Abcc8/Neto2/Lrrc	13
GO:1901653	GO:1901653	cellular response to pep	26/765	287/23210	3.56E-06	0.000146	0.000108136	Eif4ebp1/Igf1r/Tbc1d4/Appl2/Akt1/I	26
GO:0072503	GO:0072503	cellular divalent inorgan	37/765	497/23210	3.97E-06	0.000162	0.000119802	Mt2/Tgm2/Tmem38b/Mt1/Bnip3/Ch	37
GO:0032412	GO:0032412	regulation of ion transm	24/765	254/23210	4.07E-06	0.000165	0.000122005	Scn3b/Wnk2/Ank3/Dmd/Ahnak/Abc	24
GO:0051924	GO:0051924	regulation of calcium ior	25/765	272/23210	4.23E-06	0.00017	0.000125857	Tmem38b/Lgals3/Dmd/Stc2/Ahnak/	25
GO:0009991	GO:0009991	response to extracellular	32/765	401/23210	4.29E-06	0.000172	0.000126929	Eif4ebp1/Foxk1/Igf1r/Sesn1/Glul/Ap	32
GO:1902305	GO:1902305	regulation of sodium ior	11/765	61/23210	4.32E-06	0.000172	0.000126929	Scn3b/Wnk2/Ank3/Dmd/Atp1a2/Glu	11
GO:0010811	GO:0010811	positive regulation of ce	16/765	127/23210	4.49E-06	0.000178	0.00013124	Dmd/Cd36/Kdr/Myadm/Thy1/Abi3b	16
GO:0003433	GO:0003433	chondrocyte developme	7/765	22/23210	4.55E-06	0.000178	0.00013124	Col6a2/Col6a1/Col6a3/Serpinh1/Ma	7
GO:1903514	GO:1903514	release of sequestered c	7/765	22/23210	4.55E-06	0.000178	0.00013124	Tmem38b/Dmd/Nol3/Ryr1/Ank2/Ca	7
GO:1901652	GO:1901652	response to peptide	31/765	384/23210	4.78E-06	0.000186	0.000137115	Eif4ebp1/Igf1r/Tbc1d4/Appl2/Cry2/	31
GO:0001935	GO:0001935	endothelial cell prolifera	17/765	143/23210	5.07E-06	0.000195	0.000143847	Akt1/Hmox1/Stat3/Lrg1/Atoh8/Rgcc	17
GO:0019318	GO:0019318	hexose metabolic proce	22/765	223/23210	5.08E-06	0.000195	0.000143847	Foxk1/Rorc/Rora/Acacb/Sik1/Akt1/F	22
GO:0070252	GO:0070252	actin-mediated cell cont	13/765	87/23210	5.29E-06	0.000202	0.000148891	Scn3b/Kcna5/Mylk2/Atp1a2/Ank2/S	13
GO:2000649	GO:2000649	regulation of sodium ior	10/765	51/23210	5.36E-06	0.000203	0.000150012	Scn3b/Wnk2/Ank3/Dmd/Atp1a2/Glu	10
GO:0050673	GO:0050673	epithelial cell proliferatic	33/765	425/23210	5.46E-06	0.000204	0.000151024	Glul/Ctsl/Akt1/Igfbp3/Hmox1/Cebpl	33
GO:0001936	GO:0001936	regulation of endothelia	16/765	129/23210	5.51E-06	0.000204	0.000151024	Akt1/Hmox1/Stat3/Lrg1/Atoh8/Rgcc	16
GO:0071804	GO:0071804	cellular potassium ion tr	20/765	191/23210	5.53E-06	0.000204	0.000151024	Slc12a2/Tmem38b/Wnk2/Ank3/Kcn	20
GO:0071805	GO:0071805	potassium ion transmen	20/765	191/23210	5.53E-06	0.000204	0.000151024	Slc12a2/Tmem38b/Wnk2/Ank3/Kcn	20
GO:0005976	GO:0005976	polysaccharide metaboli	15/765	115/23210	5.77E-06	0.000212	0.000156669	Gaa/Akt1/Gyg/Ppp1r1a/B3gnt3/Ppp	15
GO:0010632	GO:0010632	regulation of epithelial c	22/765	225/23210	5.87E-06	0.000213	0.000157514	Glul/Mecp2/Akt1/Hmox1/Cd63/Amc	22
GO:0032868	GO:0032868	response to insulin	22/765	225/23210	5.87E-06	0.000213	0.000157514	Eif4ebp1/Igf1r/Tbc1d4/Appl2/Cry2/	22
GO:0010830	GO:0010830	regulation of myotube c	11/765	63/23210	5.99E-06	0.000215	0.000158879	Sik1/Il4ra/Myog/Naca/Trim72/Tbx1/	11

GO:0042246	GO:0042246	tissue regeneration	11/765	63/23210	5.99E-06	0.000215	0.000158879	Cdkn1a/Mustn1/Naca/Ifrd1/Serpine	11
GO:0060351	GO:0060351	cartilage development ir	10/765	52/23210	6.44E-06	0.00023	0.000169808	Col1a1/Col6a2/Col6a1/Col6a3/Hspg	10
GO:0032869	GO:0032869	cellular response to insu	20/765	193/23210	6.47E-06	0.00023	0.000169808	Eif4ebp1/Igf1r/Tbc1d4/Appl2/Akt1/I	20
GO:0031667	GO:0031667	response to nutrient leve	30/765	371/23210	6.56E-06	0.000232	0.000171181	Eif4ebp1/Foxk1/Igf1r/Sesn1/Glul/Ap	30
GO:0071375	GO:0071375	cellular response to pep	23/765	244/23210	6.69E-06	0.000234	0.000172956	Eif4ebp1/Igf1r/Tbc1d4/Appl2/Akt1/I	23
GO:0046434	GO:0046434	organophosphate catab	21/765	210/23210	6.71E-06	0.000234	0.000172956	Foxk1/Ddit4/Entpd1/Ier3/Stat3/Myc/	21
GO:0060350	GO:0060350	endochondral bone mor	12/765	76/23210	6.76E-06	0.000235	0.000173359	Col1a1/Col6a2/Col6a1/Alpl/Col6a3/	12
GO:0022898	GO:0022898	regulation of transmemb	24/765	262/23210	6.93E-06	0.000239	0.000176707	Scn3b/Wnk2/Ank3/Dmd/Ahnak/Abc	24
GO:0030308	GO:0030308	negative regulation of ce	20/765	194/23210	7.00E-06	0.00024	0.000177534	Tspyl2/Sesn1/Sema6b/Sema7a/Mec	20
GO:0006814	GO:0006814	sodium ion transport	21/765	211/23210	7.22E-06	0.000246	0.000181476	Slc12a2/Slc10a6/Scn3b/Wnk2/Ank3/	21
GO:1900542	GO:1900542	regulation of purine nuc	14/765	103/23210	7.23E-06	0.000246	0.000181476	Ddit4/Entpd1/Ier3/Pdk4/Bcl2l1/Stat3	14
GO:0001558	GO:0001558	regulation of cell growth	33/765	431/23210	7.33E-06	0.000247	0.000182585	Csf2rb/Tspyl2/Sesn1/Sema6b/Sema	33
GO:0010765	GO:0010765	positive regulation of so	9/765	42/23210	7.36E-06	0.000247	0.000182585	Scn3b/Wnk2/Ank3/Dmd/Akt1/Glrx/	9
GO:0003414	GO:0003414	chondrocyte morphoger	6/765	16/23210	7.59E-06	0.000251	0.000185306	Col6a2/Col6a1/Col6a3/Matn2/Col12	6
GO:0003429	GO:0003429	growth plate cartilage ch	6/765	16/23210	7.59E-06	0.000251	0.000185306	Col6a2/Col6a1/Col6a3/Matn2/Col12	6
GO:0090171	GO:0090171	chondrocyte morphoger	6/765	16/23210	7.59E-06	0.000251	0.000185306	Col6a2/Col6a1/Col6a3/Matn2/Col12	6
GO:0007159	GO:0007159	leukocyte cell-cell adhes	27/765	319/23210	8.25E-06	0.000271	0.000200373	Malt1/Selp/Il6ra/S100a9/Cblb/Zbtb1	27
GO:0042593	GO:0042593	glucose homeostasis	24/765	265/23210	8.40E-06	0.000275	0.000203125	Foxk1/Igf1r/Appl2/Cry2/Akt1/Pdk4/	24
GO:1903578	GO:1903578	regulation of ATP metab	13/765	91/23210	8.78E-06	0.000286	0.000211154	Ddit4/Entpd1/Ier3/Bcl2l1/Stat3/Myc/	13
GO:0033500	GO:0033500	carbohydrate homeosta	24/765	266/23210	8.96E-06	0.00029	0.000214224	Foxk1/Igf1r/Appl2/Cry2/Akt1/Pdk4/	24
GO:0003416	GO:0003416	endochondral bone gro	9/765	43/23210	9.03E-06	0.000291	0.000214964	Col6a2/Col6a1/Col6a3/Kdr/Ostn/Ma	9
GO:0045598	GO:0045598	regulation of fat cell diff	16/765	135/23210	9.94E-06	0.000319	0.000235321	Rorc/Rora/Zbtb16/Gps2/Akt1/Cebp	16
GO:0014812	GO:0014812	muscle cell migration	14/765	106/23210	1.01E-05	0.000324	0.000238955	Igfbp3/Myc/Cyp1b1/Naca/Serpine1/	14
GO:0042391	GO:0042391	regulation of membrane	33/765	439/23210	1.07E-05	0.000341	0.000251491	Scn3b/Mecp2/Bnip3/Ank3/Chrna9/I	33
GO:1903037	GO:1903037	regulation of leukocyte c	25/765	287/23210	1.08E-05	0.000341	0.000251875	Malt1/Il6ra/Cblb/Zbtb16/Lgals3/Akt	25
GO:0009166	GO:0009166	nucleotide catabolic pro	16/765	136/23210	1.09E-05	0.000342	0.000252568	Foxk1/Ddit4/Ier3/Stat3/Myc/Myog/S	16
GO:0043434	GO:0043434	response to peptide hor	27/765	324/23210	1.09E-05	0.000342	0.000252568	Eif4ebp1/Igf1r/Tbc1d4/Appl2/Cry2/A	27
GO:0006140	GO:0006140	regulation of nucleotide	14/765	107/23210	1.13E-05	0.000352	0.00026008	Ddit4/Entpd1/Ier3/Pdk4/Bcl2l1/Stat3	14
GO:0003422	GO:0003422	growth plate cartilage m	6/765	17/23210	1.14E-05	0.000353	0.000260482	Col6a2/Col6a1/Col6a3/Matn2/Col12	6
GO:0072593	GO:0072593	reactive oxygen species	25/765	288/23210	1.15E-05	0.000353	0.000260822	Gadd45a/Gpx3/Sesn1/Ddit4/Rora/S	25
GO:0010675	GO:0010675	regulation of cellular car	17/765	152/23210	1.16E-05	0.000354	0.000261452	Foxk1/Rorc/Ddit4/Rora/Acacb/Ier3/	17
GO:0031100	GO:0031100	animal organ regenerati	7/765	25/23210	1.18E-05	0.000357	0.00026354	Hmox1/Cebpb/Ezh1/Ccnd1/Vtn/Au	7
GO:0043567	GO:0043567	regulation of insulin-like	7/765	25/23210	1.18E-05	0.000357	0.00026354	Igfbp3/Trim72/Igfbp5/Bmp5/Igfbp6/	7
GO:0044843	GO:0044843	cell cycle G1/S phase tra	19/765	185/23210	1.24E-05	0.000376	0.00027767	Eif4ebp1/Rbl2/Fam107a/Fbxo31/Akt	19
GO:0032409	GO:0032409	regulation of transporter	24/765	272/23210	1.30E-05	0.000391	0.000288943	Scn3b/Wnk2/Ank3/Dmd/Ahnak/Abc	24
GO:0002063	GO:0002063	chondrocyte developme	9/765	45/23210	1.34E-05	0.0004	0.000295614	Col6a2/Col6a1/Col6a3/Col11a1/Serp	9
GO:0006109	GO:0006109	regulation of carbohydra	19/765	186/23210	1.34E-05	0.0004	0.000295623	Foxk1/Rorc/Ddit4/Rora/Acacb/Ier3/	19
GO:0071248	GO:0071248	cellular response to met	17/765	154/23210	1.37E-05	0.000407	0.000300695	Mt2/Mt1/Mecp2/Bnip3/Ank3/Akt1/I	17
GO:0032963	GO:0032963	collagen metabolic proc	14/765	109/23210	1.40E-05	0.000414	0.000305937	Il6ra/Errfi1/Rgcc/Serpine1/Col1a1/C	14
GO:0000082	GO:0000082	G1/S transition of mitoti	18/765	171/23210	1.50E-05	0.000437	0.00032268	Eif4ebp1/Rbl2/Fam107a/Fbxo31/Akt	18
GO:0043409	GO:0043409	negative regulation of M	18/765	171/23210	1.50E-05	0.000437	0.00032268	Igf1r/Wnk2/Ranbp9/Chrna9/Gps2/D	18
GO:0001933	GO:0001933	negative regulation of p	31/765	408/23210	1.60E-05	0.000465	0.000343693	Gadd45a/Igf1r/Ddit4/Wnk2/Cblb/Ra	31
GO:0098868	GO:0098868	bone growth	9/765	46/23210	1.62E-05	0.000468	0.000345291	Col6a2/Col6a1/Col6a3/Kdr/Ostn/Ma	9
GO:0045926	GO:0045926	negative regulation of g	23/765	258/23210	1.66E-05	0.000479	0.00035358	Tspyl2/Sesn1/Sema6b/Sema7a/Mec	23
GO:0055074	GO:0055074	calcium ion homeostasis	35/765	489/23210	1.67E-05	0.000479	0.000353989	Tgm2/Tmem38b/Bnip3/Chrna9/Dm	35
GO:0046165	GO:0046165	alcohol biosynthetic pro	16/765	141/23210	1.73E-05	0.000494	0.000364498	Acer2/Plcd3/Fdft1/Itpkc/Ip6k3/Dhcr	16
GO:0016052	GO:0016052	carbohydrate catabolic p	16/765	142/23210	1.89E-05	0.000537	0.000396635	Foxk1/Ddit4/Gaa/Ier3/Stat3/Myc/My	16
GO:0016525	GO:0016525	negative regulation of a	14/765	112/23210	1.92E-05	0.000544	0.000401564	Mecp2/Hgs/Cd36/Amot/Abcc8/Ada	14
GO:0072659	GO:0072659	protein localization to pl	24/765	279/23210	1.98E-05	0.000558	0.000412188	Scn3b/Ank3/Lgals3/D230025D16Rik	24
GO:0010660	GO:0010660	regulation of muscle cel	13/765	98/23210	1.99E-05	0.000558	0.000412472	Fbxo32/Igf1r/Bnip3/Pik3r1/Igfbp3/H	13
GO:0006874	GO:0006874	cellular calcium ion hom	34/765	473/23210	2.01E-05	0.000562	0.000415239	Tgm2/Tmem38b/Bnip3/Chrna9/Dm	34
GO:0060536	GO:0060536	cartilage morphogenesis	7/765	27/23210	2.05E-05	0.00057	0.000420832	Col6a2/Col6a1/Col6a3/Matn2/Col12	7
GO:0055007	GO:0055007	cardiac muscle cell differ	16/765	143/23210	2.06E-05	0.000571	0.000421693	Sik1/Myo18b/Alpk2/Mylk2/Nrap/Ag	16
GO:1901016	GO:1901016	regulation of potassium	10/765	59/23210	2.08E-05	0.000572	0.000422751	Ank3/Abcc8/Neto2/Lrrc38/Ank2/Kcr	10
GO:1904019	GO:1904019	epithelial cell apoptotic p	14/765	113/23210	2.13E-05	0.000584	0.000431021	Igf1r/Bcl2l1/Hmox1/Fas/Foxo3/Rgcc	14
GO:1902806	GO:1902806	regulation of cell cycle G	15/765	128/23210	2.14E-05	0.000585	0.00043183	Rbl2/Fam107a/Fbxo31/Akt1/Cdkn1a	15
GO:0014910	GO:0014910	regulation of smooth mu	12/765	85/23210	2.19E-05	0.000594	0.000439004	Igfbp3/Myc/Cyp1b1/Serpine1/Agt/F	12
GO:1990138	GO:1990138	neuron projection exten	19/765	193/23210	2.27E-05	0.000614	0.000453267	Sema6b/Sema7a/Mecp2/Impact/Tw	19

GO:0050873	GO:0050873	brown fat cell differentia	9/765	48/23210	2.32E-05	0.000625	0.0004614	Bnip3/Cebpb/Lrg1/Nudt7/Dusp10/F	9
GO:2000045	GO:2000045	regulation of G1/S trans	14/765	114/23210	2.35E-05	0.000631	0.000465889	Rbl2/Fam107a/Fbxo31/Akt1/Cdkn1a	14
GO:0010880	GO:0010880	regulation of release of	6/765	19/23210	2.36E-05	0.000631	0.000465889	Tmem38b/Dmd/Nol3/Ank2/Casq1/	6
GO:0055013	GO:0055013	cardiac muscle cell deve	13/765	100/23210	2.48E-05	0.00066	0.000487576	Myo18b/Alpk2/Nrap/Agt/Xirp1/Col	13
GO:0001953	GO:0001953	negative regulation of c	8/765	38/23210	2.73E-05	0.00072	0.000532058	Acer2/Fam107a/Pik3r1/Apod/Serpir	8
GO:0051235	GO:0051235	maintenance of location	26/765	322/23210	2.73E-05	0.00072	0.000532058	Lcn2/Gaa/Tmem38b/Ank3/Acacb/D	26
GO:0006979	GO:0006979	response to oxidative st	30/765	400/23210	2.79E-05	0.000732	0.000540786	Gpx3/Lcn2/Sesn1/Arnt/Bnip3/Akt1/	30
GO:0010812	GO:0010812	negative regulation of c	10/765	61/23210	2.81E-05	0.000734	0.000542036	Acer2/Fam107a/Pik3r1/Apod/Serpir	10
GO:0051961	GO:0051961	negative regulation of n	28/765	361/23210	2.82E-05	0.000734	0.000542036	Dnm3/Sema6b/Sema7a/Mecp2/App	28
GO:0050863	GO:0050863	regulation of T cell activ	25/765	304/23210	2.86E-05	0.000741	0.000547392	Rorc/Malt1/Il6ra/Cblb/Zbtb16/Lgals	25
GO:2000181	GO:2000181	negative regulation of b	14/765	116/23210	2.87E-05	0.000741	0.000547392	Mecp2/Hgs/Cd36/Amot/Abcc8/Ada	14
GO:1904064	GO:1904064	positive regulation of ca	17/765	163/23210	2.89E-05	0.000743	0.000548833	Wnk2/Ank3/Dmd/Abcc8/Agt/Thy1/	17
GO:0071407	GO:0071407	cellular response to orga	34/765	482/23210	2.96E-05	0.000755	0.000557913	Eif4ebp1/Rorc/Fbxo32/Trim63/Ddit4	34
GO:0046394	GO:0046394	carboxylic acid biosynth	28/765	362/23210	2.96E-05	0.000755	0.000557913	Foxk1/Ddit4/Glul/Acacb/Ier3/Pdk4/	28
GO:0016053	GO:0016053	organic acid biosyntheti	28/765	363/23210	3.11E-05	0.00079	0.000583146	Foxk1/Ddit4/Glul/Acacb/Ier3/Pdk4/	28
GO:0051149	GO:0051149	positive regulation of m	12/765	88/23210	3.12E-05	0.00079	0.000583146	Il4ra/Myog/Tbx1/Cth/Myf6/Smarcd3	12
GO:0006816	GO:0006816	calcium ion transport	31/765	423/23210	3.21E-05	0.000809	0.00059738	Tmem38b/Lgals3/Dmd/Catsper4/St	31
GO:0030808	GO:0030808	regulation of nucleotide	11/765	75/23210	3.33E-05	0.000832	0.000614504	Ddit4/Entpd1/Ier3/Pdk4/Bcl2l1/Stat3	11
GO:1900371	GO:1900371	regulation of purine nuc	11/765	75/23210	3.33E-05	0.000832	0.000614504	Ddit4/Entpd1/Ier3/Pdk4/Bcl2l1/Stat3	11
GO:0003413	GO:0003413	chondrocyte differentiat	7/765	29/23210	3.40E-05	0.000847	0.000625261	Col6a2/Col6a1/Col6a3/Serpinh1/Ma	7
GO:0010657	GO:0010657	muscle cell apoptotic pr	13/765	103/23210	3.41E-05	0.000847	0.000625261	Fbxo32/Igf1r/Bnip3/Pik3r1/Igfbp3/H	13
GO:0006813	GO:0006813	potassium ion transport	21/765	234/23210	3.47E-05	0.000855	0.000631234	Slc12a2/Tmem38b/Wnk2/Ank3/Kcn	21
GO:0001649	GO:0001649	osteoblast differentiatio	19/765	199/23210	3.47E-05	0.000855	0.000631234	Cebpd/Akt1/Igfbp3/Cebpb/Junb/Bn	19
GO:1904035	GO:1904035	regulation of epithelial c	12/765	89/23210	3.50E-05	0.000858	0.000633577	Igf1r/Hmox1/Foxo3/Rgcc/Serpine1/	12
GO:0014878	GO:0014878	response to electrical sti	3/765	3/23210	3.57E-05	0.000871	0.000643302	Fbxo32/Trim63/Myog	3
GO:0005996	GO:0005996	monosaccharide metabo	22/765	253/23210	3.69E-05	0.000896	0.000662128	Foxk1/Rorc/Rora/Acacb/Sik1/Akt1/F	22
GO:2000377	GO:2000377	regulation of reactive ox	19/765	200/23210	3.72E-05	0.000902	0.00066594	Gadd45a/Bnip3/Ier3/Akt1/Cdkn1a/S	19
GO:2001169	GO:2001169	regulation of ATP biosyr	10/765	63/23210	3.76E-05	0.000906	0.000669408	Ddit4/Entpd1/Ier3/Bcl2l1/Stat3/Myc	10
GO:0042445	GO:0042445	hormone metabolic proc	20/765	218/23210	3.87E-05	0.00093	0.000686955	Igf1r/Arnt/Mecp2/Ece1/Stc2/Cyp1b	20
GO:0000302	GO:0000302	response to reactive oxy	19/765	201/23210	3.99E-05	0.000955	0.000705051	Lcn2/Sesn1/Bnip3/Akt1/Apod/Hmo	19
GO:0048659	GO:0048659	smooth muscle cell proli	17/765	168/23210	4.26E-05	0.001015	0.00074999	Igf1r/Il6ra/Tgm2/Akt1/Pik3r1/Igfbp3	17
GO:0046716	GO:0046716	muscle cell cellular hom	6/765	21/23210	4.46E-05	0.001061	0.000783555	Gaa/Dmd/Lamp2/Chrna1/Lox/Cav3	6
GO:0043268	GO:0043268	positive regulation of po	9/765	52/23210	4.52E-05	0.001071	0.000790793	Wnk2/Abcc8/Lrrc38/Ank2/Kcnc1/Dp	9
GO:1903039	GO:1903039	positive regulation of leu	19/765	203/23210	4.57E-05	0.001077	0.000795353	Malt1/Il6ra/Zbtb16/Il4ra/Runx1/Prkc	19
GO:0008286	GO:0008286	insulin receptor signaling	14/765	121/23210	4.61E-05	0.001084	0.000800467	Eif4ebp1/Igf1r/Akt1/Pdk4/Pik3r1/Fo	14
GO:0055006	GO:0055006	cardiac cell developmen	13/765	106/23210	4.64E-05	0.001085	0.000801489	Myo18b/Alpk2/Nrap/Agt/Xirp1/Col	13
GO:1903522	GO:1903522	regulation of blood circu	21/765	239/23210	4.73E-05	0.001102	0.000814049	Scn3b/Gaa/Tmem38b/Sp4/Dmd/Ak	21
GO:0014909	GO:0014909	smooth muscle cell migr	12/765	92/23210	4.89E-05	0.001136	0.000839006	Igfbp3/Myc/Cyp1b1/Serpine1/Agt/F	12
GO:0050870	GO:0050870	positive regulation of T	18/765	187/23210	4.98E-05	0.001152	0.000850861	Malt1/Il6ra/Zbtb16/Il4ra/Runx1/Prkc	18
GO:0002027	GO:0002027	regulation of heart rate	12/765	93/23210	5.45E-05	0.001256	0.000927949	Scn3b/Dmd/Pik3r1/Kcna5/Agt/Ank2	12
GO:0030048	GO:0030048	actin filament-based mo	13/765	108/23210	5.65E-05	0.001292	0.000954427	Scn3b/Kcna5/Mylk2/Atp1a2/Ank2/S	13
GO:0035725	GO:0035725	sodium ion transmembr	13/765	108/23210	5.65E-05	0.001292	0.000954427	Slc12a2/Scn3b/Wnk2/Ank3/Dmd/At	13
GO:0022409	GO:0022409	positive regulation of ce	21/765	242/23210	5.66E-05	0.001292	0.000954427	Malt1/Il6ra/Ank3/Zbtb16/Il4ra/Runx	21
GO:0050768	GO:0050768	negative regulation of n	26/765	337/23210	5.88E-05	0.001337	0.000987283	Dnm3/Sema6b/Sema7a/Mecp2/App	26
GO:0003418	GO:0003418	growth plate cartilage c	6/765	22/23210	5.97E-05	0.001347	0.000994508	Col6a2/Col6a1/Col6a3/Matn2/Col12	6
GO:0010666	GO:0010666	positive regulation of ca	6/765	22/23210	5.97E-05	0.001347	0.000994508	Fbxo32/Bnip3/Igfbp3/Agt/Fndc1/Ca	6
GO:0032964	GO:0032964	collagen biosynthetic pr	9/765	54/23210	6.16E-05	0.001381	0.001019892	Il6ra/Errfi1/Rgcc/Serpine1/Col1a1/C	9
GO:0086001	GO:0086001	cardiac muscle cell actio	9/765	54/23210	6.16E-05	0.001381	0.001019892	Scn3b/Ank3/Dmd/Kcna5/Ank2/Scn	9
GO:0050678	GO:0050678	regulation of epithelial c	27/765	358/23210	6.29E-05	0.001404	0.00103732	Glul/Ctsl/Akt1/Hmox1/Stat3/Myc/Er	27
GO:0010594	GO:0010594	regulation of endothelia	16/765	157/23210	6.51E-05	0.001443	0.001065654	Glul/Mecp2/Akt1/Hmox1/Amot/Ato	16
GO:0071466	GO:0071466	cellular response to xen	16/765	157/23210	6.51E-05	0.001443	0.001065654	Eif4ebp1/Rorc/Fbxo32/Trim63/Ddit4	16
GO:0007015	GO:0007015	actin filament organizati	29/765	400/23210	6.92E-05	0.001525	0.001126475	Sptb/Fam107a/Pik3r1/Sorbs3/Prkcq	29
GO:0035690	GO:0035690	cellular response to dru	28/765	380/23210	6.95E-05	0.001525	0.001126475	Eif4ebp1/Fbxo32/Lcn2/Trim63/Ddit4	28
GO:0005978	GO:0005978	glycogen biosynthetic p	8/765	43/23210	7.00E-05	0.001525	0.001126475	Akt1/Gyg/Ppp1r3c/Irs2/Pgm2/Prkag	8
GO:0009250	GO:0009250	glucan biosynthetic proc	8/765	43/23210	7.00E-05	0.001525	0.001126475	Akt1/Gyg/Ppp1r3c/Irs2/Pgm2/Prkag	8
GO:1901381	GO:1901381	positive regulation of po	8/765	43/23210	7.00E-05	0.001525	0.001126475	Wnk2/Abcc8/Lrrc38/Ank2/Kcnc1/Dp	8
GO:0048638	GO:0048638	regulation of developme	29/765	401/23210	7.23E-05	0.001569	0.00115907	Sema6b/Sema7a/Mecp2/Acacb/Akt	29
GO:0042063	GO:0042063	gliogenesis	25/765	323/23210	7.70E-05	0.001665	0.001229826	Zcchc24/Mecp2/S100a9/Idh2/Dmd/	25

GO:0010663	GO:0010663	positive regulation of str	6/765	23/23210	7.85E-05	0.001673	0.001235936	Fbxo32/Bnip3/Igfbp3/Agt/Fndc1/Ca	6
GO:0009267	GO:0009267	cellular response to stan	15/765	143/23210	7.86E-05	0.001673	0.001235936	Sesn1/Glul/Sik1/Map1lc3b/Pdk4/Cd	15
GO:0010769	GO:0010769	regulation of cell morph	26/765	343/23210	7.87E-05	0.001673	0.001235936	Dnm3/Sema6b/Sema7a/Fbxo31/Syn	26
GO:0043467	GO:0043467	regulation of generation	14/765	127/23210	7.87E-05	0.001673	0.001235936	Ddit4/Bnip3/Ier3/Akt1/Stat3/Myc/M	14
GO:0048640	GO:0048640	negative regulation of d	14/765	127/23210	7.87E-05	0.001673	0.001235936	Sema6b/Sema7a/Mecp2/Cdkn1a/St	14
GO:1903169	GO:1903169	regulation of calcium ior	16/765	160/23210	8.17E-05	0.001731	0.001278186	Tmem38b/Dmd/Ahnak/Cacng1/Nol	16
GO:1990778	GO:1990778	protein localization to ce	26/765	344/23210	8.25E-05	0.001742	0.001286941	Scn3b/Ank3/Lgals3/D230025D16Rik	26
GO:0014741	GO:0014741	negative regulation of m	7/765	33/23210	8.31E-05	0.001744	0.001288021	Fbxo32/Trim63/Foxo1/Errfi1/Igfbp5/	7
GO:0014904	GO:0014904	myotube cell developme	8/765	44/23210	8.31E-05	0.001744	0.001288021	Dmd/Myog/Naca/Myf6/Ryr1/Klhl40/	8
GO:1901343	GO:1901343	negative regulation of va	14/765	128/23210	8.57E-05	0.001792	0.001323896	Mecp2/Hgs/Cd36/Amot/Abcc8/Ada	14
GO:0070997	GO:0070997	neuron death	29/765	405/23210	8.61E-05	0.001793	0.001324606	Ddit4/Mt1/Mecp2/Bnip3/Bcl2l1/Hm	29
GO:0010935	GO:0010935	regulation of macrophag	5/765	15/23210	8.75E-05	0.001818	0.001342799	Sema7a/Irak3/Cd36/Wnt5a/Cd74	5
GO:0001952	GO:0001952	regulation of cell-matrix	13/765	113/23210	9.06E-05	0.001875	0.001384693	Acer2/Fam107a/Dmd/Pik3r1/Apod/	13
GO:0010038	GO:0010038	response to metal ion	22/765	269/23210	9.19E-05	0.001896	0.001400509	Mt2/Mt1/Mecp2/Bnip3/Ank3/Npc1/	22
GO:0034767	GO:0034767	positive regulation of ior	17/765	179/23210	9.43E-05	0.001939	0.001432371	Wnk2/Ank3/Dmd/Abcc8/Agt/Thy1/	17
GO:0003254	GO:0003254	regulation of membrane	8/765	45/23210	9.82E-05	0.002013	0.001486761	Scn3b/Ank3/Gclm/Kdr/Myoc/Scn1b	8
GO:0035265	GO:0035265	organ growth	19/765	215/23210	9.89E-05	0.00202	0.001491897	Tmem38b/Acacb/Akt1/Pim1/Agt/Cc	19
GO:0032414	GO:0032414	positive regulation of ior	13/765	114/23210	9.92E-05	0.00202	0.001491897	Wnk2/Ank3/Dmd/Abcc8/Glrx/Lrrc38	13
GO:1901214	GO:1901214	regulation of neuron de	27/765	368/23210	9.99E-05	0.002027	0.001497249	Ddit4/Mt1/Mecp2/Bcl2l1/Hmox1/Ce	27
GO:0048660	GO:0048660	regulation of smooth mu	16/765	163/23210	0.000102	0.00206	0.001521696	Igf1r/Il6ra/Tgm2/Akt1/Pik3r1/Igfbp3	16
GO:0033002	GO:0033002	muscle cell proliferation	20/765	234/23210	0.000104	0.002096	0.001548389	Igf1r/Il6ra/Tgm2/Akt1/Pik3r1/Igfbp3	20
GO:0048545	GO:0048545	response to steroid horr	19/765	216/23210	0.000105	0.002115	0.001561945	Eif4ebp1/Fbxo32/Trim63/Ddit4/Npc	19
GO:0070838	GO:0070838	divalent metal ion transp	32/765	472/23210	0.000106	0.002121	0.001566409	Tmem38b/Lgals3/Dmd/Catsper4/St	32
GO:0051384	GO:0051384	response to glucocortico	11/765	85/23210	0.000108	0.002149	0.001586837	Eif4ebp1/Fbxo32/Trim63/Ddit4/Fam	11
GO:0010662	GO:0010662	regulation of striated mu	9/765	58/23210	0.00011	0.002185	0.00161368	Fbxo32/Bnip3/Igfbp3/Naca/Nol3/Ac	9
GO:0097193	GO:0097193	intrinsic apoptotic signal	23/765	292/23210	0.000113	0.002248	0.001660157	Ddit4/Bclaf1/Bnip3/S100a9/Chac1/Ie	23
GO:0072511	GO:0072511	divalent inorganic cation	32/765	475/23210	0.000119	0.002353	0.001737728	Tmem38b/Lgals3/Dmd/Catsper4/St	32
GO:0001508	GO:0001508	action potential	14/765	132/23210	0.00012	0.002359	0.001742407	Scn3b/Ank3/Dmd/Kcna5/Cd36/Chrn	14
GO:0014823	GO:0014823	response to activity	7/765	35/23210	0.000123	0.002427	0.001792407	Cry2/Myog/Slc4a1/Agt/Fndc5/Prkag	7
GO:0006066	GO:0006066	alcohol metabolic proce	24/765	314/23210	0.000129	0.002509	0.001853333	Mecp2/Acer2/Npc1/Plcd3/Fdft1/Cyp	24
GO:0043569	GO:0043569	negative regulation of ir	4/765	9/23210	0.000129	0.002509	0.001853333	Trim72/Igfbp5/Bmp5/Cilp	4
GO:0006817	GO:0006817	phosphate ion transport	6/765	25/23210	0.00013	0.002509	0.001853333	Cebpb/Stc2/Slc20a1/Slc37a4/Sfrp4/	6
GO:0043171	GO:0043171	peptide catabolic proces	6/765	25/23210	0.00013	0.002509	0.001853333	Chac1/Ece1/Cpq/Enpep/Ctsh/Cpe	6
GO:0085029	GO:0085029	extracellular matrix asser	6/765	25/23210	0.00013	0.002509	0.001853333	Lox/Rgcc/Agt/Col1a2/Mfap4/Antxr1	6
GO:0086019	GO:0086019	cell-cell signaling involv	6/765	25/23210	0.00013	0.002509	0.001853333	Scn3b/Kcna5/Ank2/Scn1b/Kcnn2/Sc	6
GO:0051480	GO:0051480	regulation of cytosolic ca	27/765	374/23210	0.00013	0.002509	0.001853333	Tgm2/Tmem38b/Chrna9/Dmd/Kcna	27
GO:0051155	GO:0051155	positive regulation of str	10/765	73/23210	0.000136	0.002604	0.001923177	Il4ra/Myog/Tbx1/Myf6/Mef2c/Cav3/	10
GO:1902109	GO:1902109	negative regulation of m	3/765	4/23210	0.000139	0.00266	0.001964891	Bnip3/Nol3/Acaa2	3
GO:0060349	GO:0060349	bone morphogenesis	13/765	118/23210	0.000141	0.002691	0.001987592	Col1a1/Col6a2/Col6a1/Alpl/Col6a3/	13
GO:0031102	GO:0031102	neuron projection reger	9/765	60/23210	0.000144	0.002722	0.00201019	Igf1r/Fas/Bex1/Nrep/Thy1/Ptprs/Tnc	9
GO:2000134	GO:2000134	negative regulation of G	9/765	60/23210	0.000144	0.002722	0.00201019	Rbl2/Fam107a/Fbxo31/Cdkn1a/Gpn	9
GO:0006606	GO:0006606	protein import into nucl	15/765	151/23210	0.000145	0.002746	0.002028225	Appl2/Ipo13/Cry2/Akt1/Pik3r1/Cdkn	15
GO:0043393	GO:0043393	regulation of protein bir	20/765	240/23210	0.000147	0.002761	0.002039074	Sympk/Mecp2/Cblb/Lgals3/Map1lc3	20
GO:0003417	GO:0003417	growth plate cartilage de	7/765	36/23210	0.000149	0.002796	0.00206488	Col6a2/Col6a1/Col6a3/Matn2/Col12	7
GO:0007178	GO:0007178	transmembrane recepto	26/765	357/23210	0.00015	0.00281	0.002075511	Appl2/Tmem100/Tgif1/Acvr1b/Bmp	26
GO:0031032	GO:0031032	actomyosin structure org	18/765	204/23210	0.000152	0.002835	0.002093812	Pik3r1/Sorbs3/Prkcq/Mypn/Synpo/N	18
GO:0071560	GO:0071560	cellular response to tran	18/765	204/23210	0.000152	0.002835	0.002093812	Igf1r/Appl2/Runx1/Acvr1b/Bmpr1b/	18
GO:0051282	GO:0051282	regulation of sequesteri	13/765	119/23210	0.000154	0.002853	0.002107458	Tmem38b/Dmd/Nol3/Ryr1/Ibtk/Thy	13
GO:0007009	GO:0007009	plasma membrane orga	12/765	104/23210	0.000163	0.003006	0.002219952	Sptb/S100a9/Ank3/Akt1/Trim72/Slc	12
GO:0031960	GO:0031960	response to corticostero	11/765	89/23210	0.000163	0.003006	0.002219952	Eif4ebp1/Fbxo32/Trim63/Ddit4/Fam	11
GO:0060415	GO:0060415	muscle tissue morphoge	11/765	89/23210	0.000163	0.003006	0.002219952	Mylk2/Tbx1/Ankrd1/Myf6/Col3a1/C	11
GO:0003007	GO:0003007	heart morphogenesis	22/765	280/23210	0.000164	0.003006	0.002220214	Gaa/Tmem100/Alpk2/Mylk2/Naca/F	22
GO:1903579	GO:1903579	negative regulation of A	6/765	26/23210	0.000165	0.003007	0.002220889	Ddit4/Entpd1/Ier3/Stat3/Myog/Ppif	6
GO:0051208	GO:0051208	sequestering of calcium	13/765	120/23210	0.000167	0.003051	0.002253417	Tmem38b/Dmd/Nol3/Ryr1/Ibtk/Thy	13
GO:0010934	GO:0010934	macrophage cytokine pr	5/765	17/23210	0.000171	0.003085	0.002278568	Sema7a/Irak3/Cd36/Wnt5a/Cd74	5
GO:0086012	GO:0086012	membrane depolarizatic	5/765	17/23210	0.000171	0.003085	0.002278568	Scn3b/Ank3/Scn1b/Cav3/Scn4b	5
GO:2001170	GO:2001170	negative regulation of A	5/765	17/23210	0.000171	0.003085	0.002278568	Ddit4/Entpd1/Ier3/Stat3/Myog	5
GO:0071241	GO:0071241	cellular response to inor	19/765	225/23210	0.000179	0.003232	0.002387273	Mt2/Mt1/Mecp2/Bnip3/Ank3/Ddi2/	19

GO:0032411	GO:0032411	positive regulation of tra	13/765	121/23210	0.000182	0.003269	0.00241474	Wnk2/Ank3/Dmd/Abcc8/Glrx/Lrrc38	13
GO:0010658	GO:0010658	striated muscle cell apop	9/765	62/23210	0.000186	0.003312	0.002445938	Fbxo32/Bnip3/Igfbp3/Naca/Nol3/Ac	9
GO:0014911	GO:0014911	positive regulation of sm	9/765	62/23210	0.000186	0.003312	0.002445938	Myc/Cyp1b1/Agt/Postn/Igfbp5/Lpar	9
GO:0086003	GO:0086003	cardiac muscle cell contr	9/765	62/23210	0.000186	0.003312	0.002445938	Scn3b/Kcna5/Atp1a2/Ank2/Scn1b/K	9
GO:0010976	GO:0010976	positive regulation of ne	26/765	362/23210	0.000187	0.003324	0.002454811	Igf1r/Sema7a/Mecp2/S100a9/Fbxo3	26
GO:0031346	GO:0031346	positive regulation of ce	31/765	466/23210	0.000189	0.003345	0.002470419	Igf1r/Lcn2/Dnm3/Sema7a/Mecp2/S	31
GO:0051260	GO:0051260	protein homooligomeriz	27/765	383/23210	0.000192	0.003392	0.002505096	Gpx3/Lcn2/Tgm2/Entpd1/Glul/Vwf/	27
GO:0071559	GO:0071559	response to transformin	18/765	208/23210	0.000194	0.003413	0.002520824	Igf1r/Appl2/Runx1/Acvr1b/Bmpr1b/	18
GO:0051170	GO:0051170	import into nucleus	15/765	155/23210	0.000194	0.003413	0.002520824	Appl2/Ipo13/Cry2/Akt1/Pik3r1/Cdkr	15
GO:0070371	GO:0070371	ERK1 and ERK2 cascade	24/765	323/23210	0.000197	0.003456	0.002552823	Sema7a/Wnk2/Ranbp9/Chrna9/Dm	24
GO:0001938	GO:0001938	positive regulation of en	11/765	91/23210	0.0002	0.003486	0.002574453	Akt1/Hmox1/Stat3/Lrg1/Kdr/Itga4/	11
GO:0001101	GO:0001101	response to acid chemic	26/765	364/23210	0.000204	0.003554	0.0026248	Igf1r/Sesn1/Acer2/Akt1/Pdk4/Bcl2l1	26
GO:1900543	GO:1900543	negative regulation of p	6/765	27/23210	0.000206	0.003561	0.002630202	Ddit4/Entpd1/Ier3/Stat3/Myog/Ppif	6
GO:1901018	GO:1901018	positive regulation of pc	6/765	27/23210	0.000206	0.003561	0.002630202	Abcc8/Lrrc38/Ank2/Kcnc1/Akap6/A	6
GO:0050773	GO:0050773	regulation of dendrite d	17/765	191/23210	0.000207	0.003582	0.002645914	Dnm3/Mecp2/Fbxo31/Syne1/Fat3/R	17
GO:0050708	GO:0050708	regulation of protein sec	32/765	490/23210	0.000209	0.003595	0.002655421	Arid5a/Rhbdf2/Glul/Idh2/Sec24a/Dc	32
GO:0060907	GO:0060907	positive regulation of ma	4/765	10/23210	0.00021	0.003603	0.002660892	Sema7a/Cd36/Wnt5a/Cd74	4
GO:0010831	GO:0010831	positive regulation of my	7/765	38/23210	0.000213	0.003642	0.002689734	Il4ra/Myog/Tbx1/Myf6/Cav3/Smyd1	7
GO:1901617	GO:1901617	organic hydroxy compou	19/765	229/23210	0.000225	0.003844	0.002838785	Acer2/Plcd3/Fdft1/Itpkc/Ip6k3/Dhcr	19
GO:0030809	GO:0030809	negative regulation of n	5/765	18/23210	0.00023	0.003908	0.002886138	Ddit4/Entpd1/Ier3/Stat3/Myog	5
GO:1900372	GO:1900372	negative regulation of p	5/765	18/23210	0.00023	0.003908	0.002886138	Ddit4/Entpd1/Ier3/Stat3/Myog	5
GO:0010977	GO:0010977	negative regulation of n	16/765	175/23210	0.000233	0.00394	0.002910244	Dnm3/Sema6b/Sema7a/Mecp2/Sem	16
GO:0034637	GO:0034637	cellular carbohydrate bio	10/765	78/23210	0.000237	0.004001	0.002954958	Akt1/Gyg/Fbp2/B3gnt3/Ppp1r3c/Irs	10
GO:0032233	GO:0032233	positive regulation of ac	9/765	64/23210	0.000238	0.004001	0.002954958	Sorbs3/Prkcq/Synpo/Rgcc/Lpar1/M	9
GO:1902807	GO:1902807	negative regulation of cu	9/765	64/23210	0.000238	0.004001	0.002954958	Rbl2/Fam107a/Fbxo31/Cdkn1a/Gpn	9
GO:0016051	GO:0016051	carbohydrate biosynthet	17/765	194/23210	0.00025	0.004185	0.003090731	Foxk1/Sik1/Akt1/Foxo1/Gyg/Fbp2/B	17
GO:0045663	GO:0045663	positive regulation of my	6/765	28/23210	0.000255	0.004242	0.003133339	Pik3r1/Igfbp3/Myog/Myf6/Mef2c/Sr	6
GO:0045980	GO:0045980	negative regulation of n	6/765	28/23210	0.000255	0.004242	0.003133339	Ddit4/Entpd1/Ier3/Stat3/Myog/Ppif	6
GO:0052547	GO:0052547	regulation of peptidase	28/765	411/23210	0.000259	0.0043	0.003176047	Malt1/Acer2/S100a9/Akt1/Stat3/My	28
GO:0060560	GO:0060560	developmental growth in	21/765	270/23210	0.000263	0.004356	0.003217038	Slc12a2/Sema6b/Sema7a/Mecp2/In	21
GO:0051279	GO:0051279	regulation of release of	10/765	79/23210	0.000264	0.004362	0.00322157	Tmem38b/Dmd/Nol3/Thy1/Aplnr/A	10
GO:0009410	GO:0009410	response to xenobiotic s	19/765	232/23210	0.000266	0.004381	0.003235772	Eif4ebp1/Rorc/Fbxo32/Trim63/Ddit4	19
GO:0033627	GO:0033627	cell adhesion mediated I	9/765	65/23210	0.000268	0.004415	0.003260857	Acer2/Cyp1b1/Serpine1/Fbn1/Itgb6	9
GO:0034504	GO:0034504	protein localization to nu	21/765	272/23210	0.00029	0.004762	0.003516859	Tor1aip1/Pygo1/Glul/Appl2/Ipo13/Z	21
GO:2001233	GO:2001233	regulation of apoptotic s	28/765	414/23210	0.000291	0.004762	0.003517045	Bclaf1/Bnip3/S100a9/Lgals3/Ier3/Ak	28
GO:0003009	GO:0003009	skeletal muscle contracti	7/765	40/23210	0.000297	0.004842	0.003576439	Gaa/Dmd/Mylk2/Chrna1/Casq1/Sta	7
GO:0006839	GO:0006839	mitochondrial transport	16/765	179/23210	0.000301	0.004881	0.00360497	Slc25a28/Bnip3/Ier3/Bcl2l1/Stat3/N	16
GO:1901861	GO:1901861	regulation of muscle tiss	16/765	179/23210	0.000301	0.004881	0.00360497	Myog/Naca/Pim1/Rbfox1/Tbx1/Lox	16
GO:0071548	GO:0071548	response to dexamethas	6/765	29/23210	0.000312	0.005053	0.003731955	Eif4ebp1/Fbxo32/Trim63/Ddit4/Fibi	6
GO:0046902	GO:0046902	regulation of mitochonc	8/765	53/23210	0.00032	0.005143	0.003798534	Bnip3/Ier3/Bcl2l1/Stat3/Nol3/Acaa2	8
GO:0071456	GO:0071456	cellular response to hyp	11/765	96/23210	0.000321	0.005143	0.003798534	Eif4ebp1/Rora/Bnip3/Akt1/Myc/Nol	11
GO:0014889	GO:0014889	muscle atrophy	4/765	11/23210	0.000321	0.005143	0.003798534	Fbxo32/Foxo3/Myog/Nol3	4
GO:0070278	GO:0070278	extracellular matrix cons	4/765	11/23210	0.000321	0.005143	0.003798534	Tmem38b/Rgcc/Agt/Creb3l1	4
GO:1905952	GO:1905952	regulation of lipid localiz	14/765	145/23210	0.000322	0.005143	0.003798534	Acacb/Gps2/Cry2/Akt1/Igfbp3/Runx	14
GO:1901615	GO:1901615	organic hydroxy compou	31/765	481/23210	0.000327	0.005218	0.003854113	Mecp2/Acer2/Npc1/Plcd3/Fdft1/Cyp	31
GO:0050679	GO:0050679	positive regulation of ep	17/765	199/23210	0.000337	0.005344	0.003946616	Glul/Akt1/Hmox1/Stat3/Myc/Lrg1/T	17
GO:0033692	GO:0033692	cellular polysaccharide b	9/765	67/23210	0.000339	0.005344	0.003946616	Akt1/Gyg/B3gnt3/Ppp1r3c/Irs2/Pgm	9
GO:0014735	GO:0014735	regulation of muscle atro	3/765	5/23210	0.000339	0.005344	0.003946616	Foxo3/Myog/Nol3	3
GO:0014873	GO:0014873	response to muscle activ	3/765	5/23210	0.000339	0.005344	0.003946616	Myog/Agt/Prkag3	3
GO:2000118	GO:2000118	regulation of sodium-de	3/765	5/23210	0.000339	0.005344	0.003946616	Cebpb/Stc2/Sfrp4	3
GO:0098656	GO:0098656	anion transmembrane tr	16/765	181/23210	0.000341	0.00535	0.00395151	Slc12a2/Slc7a6/Slc7a2/Akt1/Myc/Slc	16
GO:0086009	GO:0086009	membrane repolarizatio	7/765	41/23210	0.000348	0.005447	0.004023322	Kcna5/Scn1b/Kcnn2/Cav3/Akap6/Sc	7
GO:0048644	GO:0048644	muscle organ morphoge	11/765	97/23210	0.000351	0.005476	0.004044116	Mylk2/Tbx1/Ankrd1/Myf6/Col3a1/C	11
GO:0019439	GO:0019439	aromatic compound cat	31/765	483/23210	0.000351	0.005476	0.004044116	Foxk1/Gda/Ddit4/Entpd1/Ier3/Igfbp	31
GO:0032102	GO:0032102	negative regulation of re	24/765	336/23210	0.000353	0.005489	0.0040541	Sema6b/Sema7a/Rora/Appl2/Ier3/N	24
GO:0008217	GO:0008217	regulation of blood pres	17/765	200/23210	0.000357	0.005538	0.004090469	Mecp2/Ier3/Pik3r1/Hmox1/Ece1/Cd	17
GO:0035051	GO:0035051	cardiocyte differentiatio	16/765	182/23210	0.000362	0.005606	0.004140583	Sik1/Myo18b/Alpk2/Mylk2/Nrap/Ag	16
GO:0048643	GO:0048643	positive regulation of sk	6/765	30/23210	0.000379	0.005839	0.004312515	Myog/Naca/Tbx1/Myf6/Arntl/Mef2c	6

GO:0061082	GO:0061082	myeloid leukocyte cytok	6/765	30/23210	0.000379	0.005839	0.004312515	Sema7a/Hmox1/Irak3/Cd36/Wnt5a/	6
GO:0051262	GO:0051262	protein tetramerization	16/765	183/23210	0.000385	0.005916	0.004369367	Gpx3/Igf1r/Appl2/Acacb/Aldh1a1/G	16
GO:0050910	GO:0050910	detection of mechanical	5/765	20/23210	0.000394	0.006005	0.004435204	Slc12a2/Chrna9/Myc/Col11a1/Pdzd	5
GO:0051195	GO:0051195	negative regulation of co	5/765	20/23210	0.000394	0.006005	0.004435204	Ddit4/Ier3/Stat3/Myog/Hp	5
GO:0090201	GO:0090201	negative regulation of re	5/765	20/23210	0.000394	0.006005	0.004435204	Akt1/Bcl2l1/Nol3/Ppif/Igf1	5
GO:0006942	GO:0006942	regulation of striated mu	10/765	83/23210	0.000396	0.006005	0.004435204	Tmem38b/Dmd/Mylk2/Atp1a2/Smtn	10
GO:1901019	GO:1901019	regulation of calcium ion	10/765	83/23210	0.000396	0.006005	0.004435204	Dmd/Ahnak/Atp1a2/Ank2/Gnb5/Jpl	10
GO:0098742	GO:0098742	cell-cell adhesion via pla	17/765	202/23210	0.000401	0.006066	0.004480066	Selp/Cdh15/Mypn/Pvr/Fat3/Mdga1/	17
GO:0010665	GO:0010665	regulation of cardiac mu	8/765	55/23210	0.000415	0.006255	0.004619933	Fbxo32/Bnip3/Igfbp3/Nol3/Acot1/A	8
GO:0010823	GO:0010823	negative regulation of m	8/765	55/23210	0.000415	0.006255	0.004619933	Bnip3/Ier3/Akt1/Bcl2l1/Nol3/Acaa2/	8
GO:0051209	GO:0051209	release of sequestered c	12/765	115/23210	0.000418	0.006275	0.004634653	Tmem38b/Dmd/Nol3/Ryr1/Ibtk/Thy	12
GO:0062014	GO:0062014	negative regulation of sr	11/765	99/23210	0.000419	0.006286	0.004642351	Ddit4/Entpd1/Appl2/Acacb/Ier3/Sik	11
GO:0048146	GO:0048146	positive regulation of fib	9/765	69/23210	0.000423	0.006331	0.004675627	Cdkn1a/Tgif1/Myc/Serpine1/Agt/Wr	9
GO:0070588	GO:0070588	calcium ion transmembr	21/765	280/23210	0.000427	0.006374	0.00470767	Tmem38b/Dmd/Catsper4/Ahnak/Ca	21
GO:1904018	GO:1904018	positive regulation of va	17/765	204/23210	0.000449	0.006683	0.004935861	Il6ra/Lgals3/Tmem100/Hmox1/Stat3	17
GO:0010614	GO:0010614	negative regulation of ca	6/765	31/23210	0.000457	0.006759	0.00499191	Fbxo32/Trim63/Foxo1/Errfi1/Cav3/P	6
GO:0045778	GO:0045778	positive regulation of os	11/765	100/23210	0.000457	0.006759	0.00499191	Cebpd/Zbtb16/Cebpb/Bmpr1b/Cd2	11
GO:0110020	GO:0110020	regulation of actomyosin	11/765	100/23210	0.000457	0.006759	0.00499191	Pik3r1/Sorbs3/Prkcq/Synpo/Rgcc/Lp	11
GO:2001257	GO:2001257	regulation of cation chan	15/765	168/23210	0.000464	0.006846	0.005056262	Ank3/Dmd/Ahnak/Abcc8/Neto2/Lrr	15
GO:0001778	GO:0001778	plasma membrane repai	4/765	12/23210	0.000469	0.006859	0.005065651	Trim72/Rab3a/Cav3/Myh2	4
GO:0014745	GO:0014745	negative regulation of m	4/765	12/23210	0.000469	0.006859	0.005065651	Foxo1/Errfi1/Nol3/Igfbp5	4
GO:0043620	GO:0043620	regulation of DNA-temp	8/765	56/23210	0.000471	0.006859	0.005065651	Arnt/Bclaf1/Hmox1/Cebpb/Ppp1r15	8
GO:0051196	GO:0051196	regulation of coenzyme	8/765	56/23210	0.000471	0.006859	0.005065651	Ddit4/Ier3/Pdk4/Stat3/Myc/Myog/G	8
GO:0086002	GO:0086002	cardiac muscle cell actio	7/765	43/23210	0.000471	0.006859	0.005065651	Scn3b/Kcna5/Ank2/Scn1b/Kcnn2/Ca	7
GO:0046173	GO:0046173	polyol biosynthetic proc	9/765	70/23210	0.000472	0.006859	0.005065651	Acer2/Plcd3/Itpkc/Ip6k3/P2ry1/Qdp	9
GO:0051283	GO:0051283	negative regulation of se	12/765	117/23210	0.000489	0.007092	0.005237865	Tmem38b/Dmd/Nol3/Ryr1/Ibtk/Thy	12
GO:1901215	GO:1901215	negative regulation of n	19/765	244/23210	0.000499	0.007216	0.005329861	Mt1/Mecp2/Bcl2l1/Hmox1/Cebpb/S	19
GO:0097237	GO:0097237	cellular response to toxic	15/765	170/23210	0.000526	0.007596	0.005610033	Lcn2/Sesn1/Gstm2/Bnip3/Hmox1/Cy	15
GO:0002683	GO:0002683	negative regulation of in	30/765	473/23210	0.000528	0.007605	0.005616813	Cblb/Zbtb16/Lgals3/Nploc4/Gps2/A	30
GO:0050879	GO:0050879	multicellular organismal	8/765	57/23210	0.000532	0.007627	0.005632871	Gaa/Dmd/Mylk2/Chrna1/Casq1/Stac	8
GO:0050881	GO:0050881	musculoskeletal movem	8/765	57/23210	0.000532	0.007627	0.005632871	Gaa/Dmd/Mylk2/Chrna1/Casq1/Stac	8
GO:0071453	GO:0071453	cellular response to oxyg	13/765	135/23210	0.000533	0.007628	0.005633779	Eif4ebp1/Rora/Bnip3/Akt1/Myc/Foxo	13
GO:0010522	GO:0010522	regulation of calcium ion	11/765	102/23210	0.000542	0.00773	0.005708901	Tmem38b/Dmd/Nol3/Thy1/Akap5/A	11
GO:0032873	GO:0032873	negative regulation of st	7/765	44/23210	0.000544	0.00773	0.005708901	Gps2/Akt1/Per1/Foxo1/Dusp10/Dac	7
GO:0070303	GO:0070303	negative regulation of st	7/765	44/23210	0.000544	0.00773	0.005708901	Gps2/Akt1/Per1/Foxo1/Dusp10/Dac	7
GO:0072538	GO:0072538	T-helper 17 type immun	6/765	32/23210	0.000547	0.007757	0.0057291	Rorc/Malt1/Arid5a/Rora/Stat3/Prkcq	6
GO:0007204	GO:0007204	positive regulation of cy	23/765	327/23210	0.000575	0.008131	0.006005057	Tgm2/Tmem38b/Chrna9/Dmd/Cd36	23
GO:0030239	GO:0030239	myofibril assembly	9/765	72/23210	0.000582	0.008215	0.006067412	Mypn/Nrap/Lmod2/Xirp1/Casq1/Me	9
GO:1902903	GO:1902903	regulation of supramole	24/765	348/23210	0.000584	0.008215	0.006067412	Gda/Aebp1/Sptb/Mecp2/Clip1/Pik3	24
GO:0051289	GO:0051289	protein homotetrameriz	11/765	103/23210	0.000589	0.008278	0.006113999	Gpx3/Appl2/Acacb/Aldh1a1/Gnmt/	11
GO:1902307	GO:1902307	positive regulation of so	5/765	22/23210	0.000634	0.008863	0.006546153	Wnk2/Ank3/Dmd/Glrx/Atp1b2	5
GO:2000353	GO:2000353	positive regulation of en	5/765	22/23210	0.000634	0.008863	0.006546153	Foxo3/Rgcc/Itga4/Cd248/Col18a1	5
GO:1903825	GO:1903825	organic acid transmemb	11/765	104/23210	0.00064	0.008907	0.006578505	Slc7a6/Slc7a2/Akt1/Myc/Slc7a8/Agt	11
GO:1905039	GO:1905039	carboxylic acid transmer	11/765	104/23210	0.00064	0.008907	0.006578505	Slc7a6/Slc7a2/Akt1/Myc/Slc7a8/Agt	11
GO:0002082	GO:0002082	regulation of oxidative p	6/765	33/23210	0.00065	0.008929	0.006594897	Myc/Myog/Ppif/Cox7a1/Slc25a23/A	6
GO:0006084	GO:0006084	acetyl-CoA metabolic pr	6/765	33/23210	0.00065	0.008929	0.006594897	Acacb/Pdk4/Acss1/Nudt7/Acaa2/Dl	6
GO:0032970	GO:0032970	regulation of actin filam	26/765	393/23210	0.000656	0.008929	0.006594897	Sptb/Fam107a/Ctsl/Pik3r1/Sorbs3/P	26
GO:0015802	GO:0015802	basic amino acid transpo	4/765	13/23210	0.00066	0.008929	0.006594897	Slc7a2/Slc15a4/Agt/Slc38a3	4
GO:0045820	GO:0045820	negative regulation of g	4/765	13/23210	0.00066	0.008929	0.006594897	Ddit4/Ier3/Stat3/Myog	4
GO:0014854	GO:0014854	response to inactivity	3/765	6/23210	0.000662	0.008929	0.006594897	Fbxo32/Trim63/Myog	3
GO:0014870	GO:0014870	response to muscle inac	3/765	6/23210	0.000662	0.008929	0.006594897	Fbxo32/Trim63/Myog	3
GO:0014877	GO:0014877	response to muscle inac	3/765	6/23210	0.000662	0.008929	0.006594897	Fbxo32/Trim63/Myog	3
GO:0014894	GO:0014894	response to denervation	3/765	6/23210	0.000662	0.008929	0.006594897	Fbxo32/Trim63/Myog	3
GO:0035795	GO:0035795	negative regulation of m	3/765	6/23210	0.000662	0.008929	0.006594897	Bnip3/Nol3/Acaa2	3
GO:0061302	GO:0061302	smooth muscle cell-mat	3/765	6/23210	0.000662	0.008929	0.006594897	Apod/Serpine1/Vtn	3
GO:0099566	GO:0099566	regulation of postsynapt	3/765	6/23210	0.000662	0.008929	0.006594897	Synpo/Wnt5a/Fzd9	3
GO:2000323	GO:2000323	negative regulation of g	3/765	6/23210	0.000662	0.008929	0.006594897	Cry2/Per1/Arntl	3
GO:0010906	GO:0010906	regulation of glucose m	12/765	121/23210	0.000663	0.008929	0.006594897	Foxk1/Rorc/Rora/Acacb/Sik1/Akt1/F	12

GO:0042542	GO:0042542	response to hydrogen p	12/765	121/23210	0.000663	0.008929	0.006594897	Lcn2/Bnip3/Hmox1/Cyp1b1/Foxo3/I	12
GO:0010659	GO:0010659	cardiac muscle cell apop	8/765	59/23210	0.000674	0.009058	0.006690101	Fbxo32/Bnip3/Igfbp3/Nol3/Acot1/A	8
GO:0090287	GO:0090287	regulation of cellular res	20/765	271/23210	0.000709	0.009514	0.007026763	Ptp4a3/Dmd/Cd63/Hgs/Lrg1/Lox/A	20
GO:0097327	GO:0097327	response to antineoplas	9/765	74/23210	0.000714	0.009527	0.007036762	Eif4ebp1/Fbxo32/Trim63/Ddit4/Ddi2	9
GO:0006110	GO:0006110	regulation of glycolytic p	7/765	46/23210	0.000718	0.009527	0.007036762	Ddit4/Ier3/Stat3/Myc/Myog/Gpd1/I	7
GO:0030517	GO:0030517	negative regulation of a	7/765	46/23210	0.000718	0.009527	0.007036762	Sema6b/Sema7a/Sema3g/Ifrd1/Ptp	7
GO:0030811	GO:0030811	regulation of nucleotide	7/765	46/23210	0.000718	0.009527	0.007036762	Ddit4/Ier3/Stat3/Myc/Myog/Gpd1/I	7
GO:0098754	GO:0098754	detoxification	7/765	46/23210	0.000718	0.009527	0.007036762	Sesn1/Mt2/Mt1/Gstm2/Cd36/Pon3/	7
GO:0045666	GO:0045666	positive regulation of ne	29/765	461/23210	0.000739	0.009782	0.007224711	Igf1r/Sema7a/Mecp2/S100a9/Fbxo3	29
GO:0050727	GO:0050727	regulation of inflammato	24/765	354/23210	0.000742	0.009792	0.007232082	Tgm2/Sema7a/Rora/Appl2/Slc7a2/S	24
GO:0060485	GO:0060485	mesenchyme developme	20/765	272/23210	0.000743	0.009792	0.007232082	Sema6b/Sema7a/Tmem100/Myc/Se	20
GO:0048661	GO:0048661	positive regulation of sm	11/765	106/23210	0.000752	0.00989	0.007304835	Igf1r/Il6ra/Tgm2/Akt1/Hmox1/Myc/	11
GO:0009612	GO:0009612	response to mechanical	15/765	176/23210	0.000755	0.009898	0.007310763	Slc12a2/Chrna9/Dmd/Akt1/Myc/Jun	15
GO:0016202	GO:0016202	regulation of striated mu	15/765	176/23210	0.000755	0.009898	0.007310763	Myog/Naca/Pim1/Rbfox1/Tbx1/Lox/	15
GO:0032526	GO:0032526	response to retinoic acic	10/765	90/23210	0.000758	0.009907	0.007317426	Acer2/Runx1/Twf2/Tbx1/Gdap1/Me	10
GO:0005201	GO:0005201	extracellular matrix struc	42/758	140/22710	1.48E-28	1.95E-25	1.58E-25	Vwf/Col1a1/Col1a2/Postn/Col6a2/C	42
GO:0030020	GO:0030020	extracellular matrix struc	18/758	38/22710	3.89E-17	2.57E-14	2.08E-14	Col1a1/Col1a2/Col6a2/Col3a1/Col6	18
GO:0005518	GO:0005518	collagen binding	17/758	69/22710	7.80E-11	3.43E-08	2.78E-08	Aebp1/Vwf/Sparc/Abi3bp/Lum/Serp	17
GO:0008201	GO:0008201	heparin binding	23/758	155/22710	2.05E-09	6.75E-07	5.47E-07	Selp/Gpnmb/Anxa4/Postn/Col5a1/F	23
GO:0050839	GO:0050839	cell adhesion molecule b	29/758	241/22710	2.60E-09	6.87E-07	5.56E-07	Sema7a/Vwf/Ank3/Prom1/Gpnmb/	29
GO:1901681	GO:1901681	sulfur compound bindin	28/758	250/22710	2.39E-08	5.25E-06	4.25E-06	Selp/Gstm2/Acacb/Gpnmb/Anxa4/S	28
GO:0001968	GO:0001968	fibronectin binding	10/758	31/22710	3.80E-08	7.16E-06	5.80E-06	Igfbp3/Ssc5d/Itga4/Tnc/Igfbp5/Myc	10
GO:0005539	GO:0005539	glycosaminoglycan bind	24/758	208/22710	1.36E-07	2.24E-05	1.81E-05	Selp/Gpnmb/Anxa4/Postn/Col5a1/F	24
GO:0050998	GO:0050998	nitric-oxide synthase bir	8/758	22/22710	3.13E-07	4.59E-05	3.72E-05	Dnm3/Dmd/Calm2/Cd74/Calm3/Dn	8
GO:0050840	GO:0050840	extracellular matrix bind	12/758	59/22710	4.65E-07	6.13E-05	4.96E-05	Lgals3/Ssc5d/Sparc/Cd248/Nid1/Vtr	12
GO:0005178	GO:0005178	integrin binding	17/758	128/22710	1.28E-06	0.000154	0.000124398	Sema7a/Vwf/Gpnmb/Col3a1/Kdr/Fb	17
GO:0019838	GO:0019838	growth factor binding	18/758	147/22710	2.09E-06	0.000229	0.000185896	Osmr/Igf1r/Il6ra/Igfbp3/Acvr1b/Bmp	18
GO:0048407	GO:0048407	platelet-derived growth	5/758	12/22710	2.66E-05	0.002702	0.002188954	Col1a1/Col1a2/Col3a1/Col6a1/Col5a	5
GO:0016215	GO:0016215	acyl-CoA desaturase act	4/758	7/22710	3.98E-05	0.003746	0.003034741	Scd1/Fads2/Fads1/Scd2	4
GO:0044325	GO:0044325	ion channel binding	15/758	136/22710	5.06E-05	0.004448	0.003603084	Scn3b/Ank3/Abcc8/Lrrc38/Calm2/A	15
GO:0005496	GO:0005496	steroid binding	13/758	108/22710	6.41E-05	0.005282	0.004278931	Rorc/Rora/Npc1/Gramd1b/Apod/Pr	13
GO:0016717	GO:0016717	oxidoreductase activity,	4/758	8/22710	7.74E-05	0.006007	0.004866053	Scd1/Fads2/Fads1/Scd2	4
GO:0002020	GO:0002020	protease binding	15/758	146/22710	0.000114	0.008365	0.006776218	Lcn2/Malt1/Vwf/Fas/Nol3/Serpine1/	15
GO:0008179	GO:0008179	adenylate cyclase bindin	5/758	16/22710	0.000131	0.009123	0.007389755	Adcy2/Akap5/Calm2/Calm3/Akap6	5
GO:0062023	GO:0062023	collagen-containing ext	69/774	359/23436	4.96E-33	3.36E-30	2.65E-30	Tgm2/Entpd1/Vwf/Lgals3/Ctsl/Runx	69
GO:0031012	GO:0031012	extracellular matrix	75/774	475/23436	6.12E-30	2.08E-27	1.64E-27	Tgm2/Entpd1/Vwf/Lgals3/Ctsl/Runx	75
GO:0030016	GO:0030016	myofibril	43/774	212/23436	6.22E-22	1.41E-19	1.11E-19	Fbxo32/Scn3b/Ank3/Rpl6/Dmd/My	43
GO:0043292	GO:0043292	contractile fiber	44/774	226/23436	1.15E-21	1.95E-19	1.54E-19	Fbxo32/Trim63/Scn3b/Ank3/Rpl6/D	44
GO:0030017	GO:0030017	sarcomere	39/774	189/23436	2.74E-20	3.71E-18	2.93E-18	Fbxo32/Scn3b/Ank3/Rpl6/Dmd/My	39
GO:0044449	GO:0044449	contractile fiber part	40/774	203/23436	5.23E-20	5.91E-18	4.66E-18	Fbxo32/Scn3b/Ank3/Rpl6/Dmd/My	40
GO:0031674	GO:0031674	I band	31/774	142/23436	3.91E-17	3.54E-15	2.79E-15	Fbxo32/Scn3b/Ank3/Dmd/Myo18b/	31
GO:0042383	GO:0042383	sarcolemma	33/774	163/23436	4.17E-17	3.54E-15	2.79E-15	Igf1r/Car4/Ank3/Dmd/Fas/Prkcq/Ah	33
GO:0030018	GO:0030018	Z disc	27/774	130/23436	1.57E-14	1.18E-12	9.34E-13	Fbxo32/Scn3b/Ank3/Dmd/Myo18b/	27
GO:0005604	GO:0005604	basement membrane	23/774	107/23436	6.50E-13	4.41E-11	3.47E-11	Entpd1/Runx1/Serpinf1/Loxl2/Col5a	23
GO:0005581	GO:0005581	collagen trimer	20/774	81/23436	1.32E-12	8.13E-11	6.42E-11	Lox/Col1a1/Col1a2/Col6a2/Col3a1/	20
GO:0044420	GO:0044420	extracellular matrix com	15/774	51/23436	5.59E-11	3.16E-09	2.49E-09	Col1a1/Col1a2/Col3a1/Mfap4/Col5a	15
GO:0005583	GO:0005583	fibrillar collagen trimer	8/774	11/23436	2.06E-10	1.00E-08	7.88E-09	Col1a1/Col1a2/Col3a1/Col5a1/Lum/	8
GO:0098643	GO:0098643	banded collagen fibril	8/774	11/23436	2.06E-10	1.00E-08	7.88E-09	Col1a1/Col1a2/Col3a1/Col5a1/Lum/	8
GO:0098644	GO:0098644	complex of collagen trim	9/774	16/23436	4.15E-10	1.88E-08	1.48E-08	Col1a1/Col1a2/Col3a1/Col5a1/Lum/	9
GO:0014704	GO:0014704	intercalated disc	14/774	62/23436	1.09E-08	4.49E-07	3.54E-07	Ank3/Kcna5/Des/Nrap/Slc4a1/Atp1a	14
GO:0044291	GO:0044291	cell-cell contact zone	16/774	83/23436	1.13E-08	4.49E-07	3.54E-07	Ank3/Kcna5/Ahnak/Des/Nrap/Slc4a	16
GO:0030315	GO:0030315	T-tubule	14/774	70/23436	5.69E-08	2.14E-06	1.69E-06	Igf1r/Ank3/Ahnak/Cacng1/Atp1a2/F	14
GO:0005911	GO:0005911	cell-cell junction	38/774	477/23436	6.06E-07	2.06E-05	1.62E-05	Abcb1b/Sympk/Ank3/Akt1/Pik3r1/C	38
GO:0005614	GO:0005614	interstitial matrix	7/774	17/23436	6.07E-07	2.06E-05	1.62E-05	Abi3bp/Tnc/Ccdc80/Ecm2/Smoc2/C	7
GO:0031672	GO:0031672	A band	9/774	40/23436	4.87E-06	0.000157	0.000123883	Rpl6/Lmod2/Obscn/Klhl40/Ank2/My	9
GO:0016942	GO:0016942	insulin-like growth facto	4/774	5/23436	5.75E-06	0.000169	0.000133654	Igfbp3/Igfbp5/Igfbp6/Igf1	4
GO:0036454	GO:0036454	growth factor complex	4/774	5/23436	5.75E-06	0.000169	0.000133654	Igfbp3/Igfbp5/Igfbp6/Igf1	4
GO:0015629	GO:0015629	actin cytoskeleton	36/774	490/23436	7.52E-06	0.000212	0.000167471	Sptb/Filip1/Fam107a/Myo18b/Ahna	36

GO:0016528	GO:0016528	sarcoplasm	11/774	84/23436	9.83E-05	0.00258	0.002034939	Car4/Ank3/Nol3/Flnc/Ryr1/Thbs4/Jp	11
GO:1902495	GO:1902495	transmembrane transpo	24/774	308/23436	9.89E-05	0.00258	0.002034939	Scn3b/Chrna9/Catsper4/Kcna5/Cacn	24
GO:1990351	GO:1990351	transporter complex	24/774	315/23436	0.00014	0.003273	0.002581627	Scn3b/Chrna9/Catsper4/Kcna5/Cacn	24
GO:0005588	GO:0005588	collagen type V trimer	3/774	4/23436	0.00014	0.003273	0.002581627	Col5a1/Col5a2/Col5a3	3
GO:0042567	GO:0042567	insulin-like growth facto	3/774	4/23436	0.00014	0.003273	0.002581627	Igfbp3/Igfbp5/Igf1	3
GO:0016529	GO:0016529	sarcoplasmic reticulum	10/774	75/23436	0.000174	0.003924	0.003094866	Car4/Ank3/Nol3/Ryr1/Thbs4/Jph2/C	10
GO:0045121	GO:0045121	membrane raft	26/774	364/23436	0.000211	0.004611	0.003636727	Igf1r/Cblb/Adcy2/Npc1/Dmd/Hmox	26
GO:0098857	GO:0098857	membrane microdomair	26/774	365/23436	0.00022	0.004663	0.003677658	Igf1r/Cblb/Adcy2/Npc1/Dmd/Hmox	26
GO:0034702	GO:0034702	ion channel complex	22/774	293/23436	0.000319	0.006557	0.005171167	Scn3b/Chrna9/Catsper4/Kcna5/Cacn	22
GO:0031594	GO:0031594	neuromuscular junction	11/774	97/23436	0.000357	0.00683	0.005386704	Ank3/Prkcq/Des/Cdh15/Chrna1/Pos	11
GO:0043230	GO:0043230	extracellular organelle	12/774	113/23436	0.000362	0.00683	0.005386704	Car4/Prom1/Ahnak/Cd63/Lamp2/Se	12
GO:0098589	GO:0098589	membrane region	26/774	377/23436	0.000363	0.00683	0.005386704	Igf1r/Cblb/Adcy2/Npc1/Dmd/Hmox	26
GO:0034703	GO:0034703	cation channel complex	18/774	219/23436	0.000374	0.006858	0.005408555	Scn3b/Catsper4/Kcna5/Cacng1/Abc	18
GO:0001917	GO:0001917	photoreceptor inner seg	8/774	56/23436	0.000477	0.008512	0.006713504	Dnm3/Lrrc30/Gnb5/Cib2/Dnm1/Pkr	8
GO:0044304	GO:0044304	main axon	10/774	86/23436	0.000536	0.009317	0.007347919	Ank3/Thy1/Myoc/Tiam1/Kcnc1/Scn1	10
GO:0030659	GO:0030659	cytoplasmic vesicle mem	22/774	306/23436	0.000576	0.009762	0.007698938	Selp/Car4/Appl2/D230025D16Rik/Se	22

Table S3-KEGG

	ID	Description	GeneRatio	BgRatio	pvalue	p.adjust	qvalue	geneID	Coun	Regulation
1	mmu04630	JAK-STAT sign	14/188	86/4348	1.44E-05	0.003723696	0.00329919	12984/18414/	14	up
2	mmu04060	Cytokine-cytol	12/188	81/4348	0.000154374	0.016915507	0.014987119	12984/18414/	12	up
3	mmu04066	HIF-1 signaling	12/188	83/4348	0.000195933	0.016915507	0.014987119	13685/16001/	12	up
4	mmu04931	Insulin resistar	12/188	87/4348	0.000308244	0.019958767	0.017683443	210789/10070	12	up
5	mmu04068	FoxO signaling	13/188	104/4348	0.000466516	0.024165546	0.021410644	13197/67731/	13	up
6	mmu05221	Acute myeloid	8/188	51/4348	0.001353674	0.058433582	0.051772082	13685/235320	8	up
7	mmu04140	Autophagy - a	13/188	121/4348	0.001961086	0.072560175	0.064288227	16001/74747/	13	up
8	mmu04913	Ovarian steroi	5/188	24/4348	0.003111297	0.10072824	0.089245097	16001/210044	5	up
9	mmu05202	Transcriptiona	12/188	118/4348	0.004583765	0.114837077	0.10174551	13197/16001/	12	up
10	mmu04115	p53 signaling	7/188	49/4348	0.00464206	0.114837077	0.10174551	13197/140742	7	up
11	mmu04710	Circadian rhytl	5/188	27/4348	0.0053193	0.114837077	0.10174551	19885/19883/	5	up
12	mmu04218	Cellular senesc	12/188	122/4348	0.005996745	0.114837077	0.10174551	13685/13197/	12	up
13	mmu04211	Longevity regu	9/188	78/4348	0.006009129	0.114837077	0.10174551	13685/16001/	9	up
14	mmu04668	TNF signaling	9/188	79/4348	0.006534898	0.114837077	0.10174551	26410/11651/	9	up
15	mmu05169	Epstein-Barr v	13/188	140/4348	0.006979376	0.114837077	0.10174551	13197/12495/	13	up
16	mmu01521	EGFR tyrosine	8/188	67/4348	0.007688848	0.114837077	0.10174551	13685/16001/	8	up
17	mmu04213	Longevity regu	7/188	54/4348	0.007980955	0.114837077	0.10174551	16001/210044	7	up
18	mmu04920	Adipocytokine	7/188	54/4348	0.007980955	0.114837077	0.10174551	100705/11651	7	up
19	mmu04910	Insulin signalir	11/188	114/4348	0.009651946	0.13157127	0.116571985	13685/208650	11	up
20	mmu05200	Pathways in ca	25/188	358/4348	0.010409619	0.134804568	0.119436683	12984/13197/	25	up
21	mmu05204	Chemical carci	4/188	21/4348	0.011361486	0.135351599	0.119921352	11863/14863/	4	up
22	mmu04152	AMPK signalin	10/188	102/4348	0.01201964	0.135351599	0.119921352	13685/16001/	10	up
23	mmu04210	Apoptosis	10/188	102/4348	0.01201964	0.135351599	0.119921352	12984/13197/	10	up
24	mmu04216	Ferroptosis	5/188	34/4348	0.014362445	0.154994716	0.137325129	67443/15368/	5	up
25	mmu03050	Proteasome	5/188	38/4348	0.022554101	0.202279935	0.179219776	19185/57296/	5	up
26	mmu04659	Th17 cell differ	7/188	66/4348	0.022753242	0.202279935	0.179219776	19885/16194/	7	up
27	mmu05213	Endometrial ca	6/188	52/4348	0.023483252	0.202279935	0.179219776	13197/11651/	6	up
28	mmu04978	Mineral absorp	4/188	26/4348	0.023997058	0.202279935	0.179219776	17750/17748/	4	up
29	mmu05321	Inflammatory I	4/188	26/4348	0.023997058	0.202279935	0.179219776	19885/19883/	4	up
30	mmu04933	AGE-RAGE sig	8/188	82/4348	0.02428416	0.202279935	0.179219776	11651/18708/	8	up
31	mmu04660	T cell receptor	7/188	67/4348	0.024510755	0.202279935	0.179219776	240354/20865	7	up
32	mmu00480	Glutathione m	5/188	39/4348	0.024992115	0.202279935	0.179219776	14778/14863/	5	up
33	mmu04923	Regulation of I	5/188	40/4348	0.027593386	0.211459471	0.187352834	210044/11651	5	up
34	mmu01524	Platinum drug	6/188	54/4348	0.027759158	0.211459471	0.187352834	14863/11651/	6	up
35	mmu04550	Signaling path	8/188	85/4348	0.029401758	0.217573011	0.192769423	16001/11651/	8	up
36	mmu05220	Chronic myelo	7/188	71/4348	0.032471969	0.227648879	0.201696629	13197/11651/	7	up
37	mmu04137	Mitophagy - a	6/188	56/4348	0.032521268	0.227648879	0.201696629	12176/12048/	6	up
38	mmu04151	PI3K-Akt signa	16/188	228/4348	0.036183423	0.246618591	0.218503771	18414/13685/	16	up
39	mmu05223	Non-small cell	6/188	58/4348	0.03778622	0.250939256	0.222331875	13197/11651/	6	up
40	mmu04935	Growth hormc	8/188	90/4348	0.039506286	0.255803204	0.226641327	210044/11651	8	up
41	mmu05206	MicroRNAs in	10/188	126/4348	0.044711075	0.282443134	0.250244273	18669/74747/	10	up
42	mmu04974	Protein digesti	20/197	47/4348	1.71E-15	4.11E-13	3.84E-13	12842/12843/	20	down
43	mmu04512	ECM-receptor	17/197	59/4348	3.73E-10	4.49E-08	4.20E-08	12842/12843/	17	down
44	mmu04510	Focal adhesior	22/197	157/4348	1.53E-06	0.000122804	0.000114786	12842/12843/	22	down
45	mmu04151	PI3K-Akt signa	24/197	228/4348	7.48E-05	0.004505977	0.004211745	12842/12843/	24	down
46	mmu05205	Proteoglycans	18/197	158/4348	0.000233692	0.011263934	0.01052842	12842/12843/	18	down
47	mmu05165	Human papillc	21/197	233/4348	0.001718319	0.069019154	0.064512335	12842/12843/	21	down
48	mmu00100	Steroid biosyn	4/197	13/4348	0.002116625	0.072872388	0.06811396	13360/74754/	4	down
49	mmu00260	Glycine, serine	5/197	22/4348	0.002542056	0.076579439	0.071578947	11655/27364/	5	down
50	mmu04261	Adrenergic sig	12/197	109/4348	0.003489147	0.090673384	0.084752584	26427/319734	12	down
51	mmu00790	Folate biosyntl	4/197	15/4348	0.003762381	0.090673384	0.084752584	11647/110391	4	down
52	mmu01040	Biosynthesis o	4/197	16/4348	0.004840978	0.106061418	0.099135809	20249/56473/	4	down
53	mmu00010	Glycolysis / Gl	6/197	38/4348	0.006574402	0.129727184	0.121256245	235339/72157	6	down
54	mmu00230	Purine metabc	10/197	90/4348	0.006997732	0.129727184	0.121256245	18577/11534/	10	down
55	mmu05410	Hypertrophic c	8/197	65/4348	0.008430723	0.141434698	0.132199281	16420/16401/	8	down
56	mmu04922	Glucagon sign	9/197	79/4348	0.00880299	0.141434698	0.132199281	26427/12314/	9	down
57	mmu00980	Metabolism of	4/197	22/4348	0.015731717	0.230699497	0.215635259	14860/12409/	4	down

58	mmu04260	Cardiac muscle	7/197	59/4348	0.016273409	0.230699497	0.215635259	66445/319734	7	down
59	mmu04371	Apelin signalin	10/197	103/4348	0.017357321	0.232395246	0.217220278	12443/23796/	10	down
60	mmu05031	Amphetamine	5/197	36/4348	0.021791997	0.276414278	0.258364951	26427/53623/	5	down
61	mmu00760	Nicotinate anc	4/197	26/4348	0.027936561	0.330085283	0.308531341	64384/69564/	4	down
62	mmu04911	Insulin secretic	6/197	52/4348	0.028762618	0.330085283	0.308531341	26427/19339/	6	down
63	mmu05414	Dilated cardior	7/197	68/4348	0.032924465	0.360672553	0.337121321	16420/16401/	7	down
64	mmu01212	Fatty acid met	5/197	41/4348	0.036146391	0.378751312	0.354019571	52538/20249/	5	down
65	mmu04964	Proximal tubul	3/197	17/4348	0.038960615	0.381424149	0.356517877	76257/27376/	3	down
66	mmu04713	Circadian entr	6/197	56/4348	0.03956682	0.381424149	0.356517877	53623/66066/	6	down
67	mmu05146	Amoebiasis	6/197	57/4348	0.042621144	0.395065219	0.36926821	12842/12843/	6	down
68	mmu04020	Calcium signal	9/197	105/4348	0.047098393	0.420396771	0.39294566	105675/12314	9	down

Table S4

GEO_accession	Journal	Test_assigned	Sample_size	Mouse_strain	Model	Cancer type	Muscle type	Body weight loss	Anorexia
GSE133524	Cell report	Test1	4	C57BL/6J	KPP(KrasLSL-G12D/+, Ptenf/f , Ptf1aER-Cre/+)	Pancreatic Cancer	Tibalis anterior	~30%	Not available
			4	C57BL/6J	KPC(Kras+/LSL-G12D, Trp53+/R270H, Pdx1+/Cre)	Pancreatic Cancer	Tibalis anterior	~25%	Not available
			4	CD2F1	C26 xenograft	Colon Cancer	Tibalis anterior	~20%	Not available
			4	C57BL/6J	LLC xenograft	Lung Cancer	Tibalis anterior	~20%	Not available
GSE138464	Embo molecular medicine	Test2	10	CD2F1	C26 xenograft	Colon Cancer	Gastrocnemius	~20%	Observed
GSE107470	Proceedings of the National Academy of Sciences	Test3	10	~75% C57Bl/6, ~25% FVB/n and 129SvEv	KrasG12D/+;Lkb1f/f	Lung Cancer	Gastrocnemius	~30%	Observed
GSE142455	JCI insight	Test4	4	CD2F1	C26 splenic xenograft	Colon Cancer	Tibalis anterior	~4g*	Not available
			4	CD2F1	C26 subcutaneous xenograft	Colon Cancer	Tibalis anterior	~4g*	Not available
GSE137985	Journal of Cachexia, Sarcopenia and Muscle	Test5	34	NOD.Cg-PrkdcscidIl2rgtm1Wjl/SzJ	PDX	Pancreatic Cancer	Tibalis anterior	~25%	Not available
Na	Na	Test6	6	Babl/c	C26 xenograft	Colon Cancer	Gastrocnemius	~25%	Not siginificant
								*The original data of the whole body weight is not available	
